# Prediction of disease resilience of pigs using multi-omics data

**DOI:** 10.1186/s40104-026-01416-9

**Published:** 2026-06-02

**Authors:** Yulu Chen, Steven Lonergan, Kyu-Sang Lim, Jian Cheng, Elda Dervishi, Michael K. Dyck, Edward Steadham, PigGen Canada, Frederic Fortin, John C. S. Harding, Graham S. Plastow, Jack C. M. Dekkers

**Affiliations:** 1 Department of Animal Science, Iowa State University, 239D Kildee Hall, Ames, IA 50011 USA; 2 Channing Division of Network Medicine, Department of Medicine, Brigham and Women’s Hospital and Harvard Medical School, Boston, MA 02115 USA; 3 Department of Animal Resources Science, Kongju National University, Chungnam, 32439 Republic of Korea; 4 Department of Agriculture, Food and Nutritional Science, University of Alberta, Edmonton, AB T6G 2R3 Canada; 5 PigGen Canada Research Consortium, Guelph, ON N1H4G8 Canada; 6 Centre de Développement du Porc du Québec Inc., Québec City, G1V 4M6 Canada; 7 Department of Large Animal Clinical Science, University of Saskatchewan, Saskatoon, SK S7N 5A2 Canada

**Keywords:** Disease resilience, Genomics, Metabolomics, Multi-omics, Natural disease challenge model, Phenotype prediction, Pigs, Proteomics, Transcriptomics

## Abstract

**Background:**

Genomic prediction is widely used in pig breeding, but phenotypic prediction of complex traits such as disease resilience remains limited because genotypes alone do not capture infection-induced regulatory responses, environmental and management effects, or their interactions. Blood molecular profiles measured in young healthy pigs reflect both genetic and non-genetic influences and may improve prediction of performance under disease challenge. We evaluated whether integrating multiple blood-based omics layers with genomic data improves prediction of production and disease resilience phenotypes in pigs exposed to a polymicrobial disease challenge.

**Results:**

Data were from 836 healthy pigs from 15 batches with transcriptomic, proteomic, and metabolomic profiles measured in blood collected at ~27 days of age, before transfer into a natural polymicrobial disease challenge at ~40 days of age. Pigs were also genotyped using a commercial 650 K marker array. We analyzed 21 traits related to growth, health scores, antibiotic treatments, mortality, feed efficiency, and carcass traits using best linear unbiased prediction (BLUP) animal models with random animal effects based on relationship matrices constructed from genomic (G), transcriptomic (T), proteomic (P), and metabolomic (M) data. Across traits, G-BLUP explained the largest proportion of phenotypic variance for most traits. However, T-, P-, or M-BLUP explained similar or greater variance than G-BLUP for several growth and health traits recorded before challenge. Adding T and/or M to G-BLUP generally increased variance explained and improved prediction accuracy for pre-challenge growth rate and health scores, and for mortality and carcass weight after challenge. Models combining G, T, and M often yielded the highest accuracies, whereas adding P did not consistently improve accuracy. For later grow-finish traits, gains from multi-omics were smaller and less consistent.

**Conclusions:**

Blood multi-omics profiles from healthy young pigs can improve prediction of performance and disease resilience beyond genomic data alone. Gains were greatest for traits recorded before challenge and for some resilience traits expressed soon after pathogen exposure, suggesting that pre-challenge molecular profiles capture latent resilience potential. These findings support the use of pre-challenge blood multi-omics as biomarkers for precision management and as a basis for breeding and management strategies targeting disease resilience in pigs.

**Supplementary Information:**

The online version contains supplementary material available at 10.1186/s40104-026-01416-9.

## Background

Improving animal health is a key component of sustainable pig production, as infectious diseases reduce growth and feed efficiency, increase mortality and treatment costs, and raise concerns about animal welfare and antimicrobial use [1–6]. In commercial systems, pigs are exposed to heterogeneous and often polymicrobial disease pressure, making it difficult to target individual pathogens. Accordingly, breeding and management strategies increasingly focus on disease resilience, defined as the ability to maintain health and performance under challenge and to recover following disease and other stressors, rather than pathogen-specific resistance [1–5, 7]. A key practical goal is to develop prediction tools for resilience-related outcomes using information that can be collected early in life, including on young, clinically healthy pigs.

In this context, genomic prediction using genotypes at genetic markers across the genome has been widely adopted in pig breeding programs and has substantially increased the rate of genetic gain for many production and reproduction traits [8–10]. However, improving resilience remains challenging because selection candidates are typically raised in high-health environments and resilience phenotypes are complex and strongly influenced by environment and management; consequently, prediction accuracies for resilience-related outcomes are often lower than for classical production traits. In addition, the ability to identify which pigs are more resilient to disease at an early age would be beneficial for management in commercial swine production. These limitations suggest that marker genotypes alone may be insufficient to capture the downstream biology and short-term physiological variation that influence resilience, motivating the use of molecular endophenotypes.

High-throughput profiling of transcripts, proteins, and metabolites provides intermediate molecular readouts that integrate genetic predisposition and early-life environment and may therefore complement marker genotypes for prediction of complex traits [11–13], capturing baseline physiological and immune states not fully represented by genotype alone. A practical approach for incorporating high-dimensional omics into prediction is to represent each omics layer by a relationship (kernel) matrix computed from standardized features and fit these kernels as random effects in linear mixed models (multi-kernel best linear unbiased prediction, BLUP). Previous studies in livestock and crops suggest that adding single omics layers can improve prediction for some traits, although gains tend to be trait- and context-dependent [11, 14, 15].

Despite this progress, it remains unclear to what extent blood-based multi-omics profiles collected from clinically healthy piglets can improve prediction of subsequent resilience-related outcomes under an industry-relevant polymicrobial disease challenge. In particular, few studies have evaluated the joint use of multiple blood omics layers together with genomics, and the traits and production phases that benefit most from multi-omics integration are not well established.

To address these questions in an industry-relevant polymicrobial challenge setting, we leveraged data from the natural disease challenge model (NDCM) described by Putz et al. [16], in which enabling collection of resilience-related traits across production phases [17]. We evaluated genomic, transcriptomic, proteomic, and metabolomic information measured in blood collected from young, clinically healthy pigs for prediction of resilience-related outcomes and performance under a polymicrobial disease challenge. Specifically, we compared single- and multi-kernel BLUP models and assessed whether integrating omics layers with genomics improves leave-one-batch-out prediction across production phases and trait domains. This study provides a systematic evaluation of the relative and complementary predictive value of blood transcriptomic, proteomic, and metabolomic layers, alone and in combination with genomics, for resilience-related traits in an industry-relevant natural disease challenge setting.

## Methods

### Study design

**Fig. 1:**
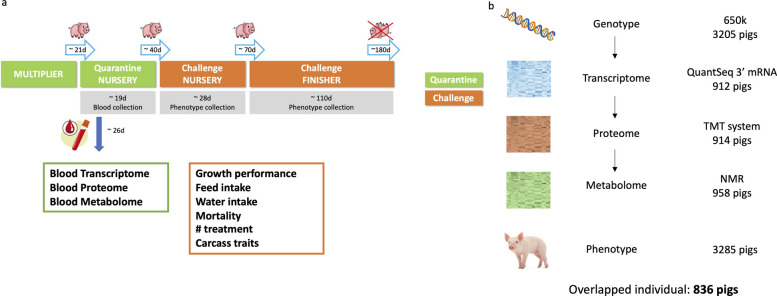
Overview of the data generation. **a** The natural disease challenge model. **b** Summary of the omics data used and associated sample sizes

Data were collected in the NDCM described previously [16, 17], which is schematically illustrated in Fig. 1a. Briefly, every 3 weeks, a batch of 60 or 75 recently weaned ~21 day-old Yorkshire × Landrace F1 barrows originating from a single member breeding company (“company”) of the PigGen Canada research consortium entered the NDCM; batches from the seven companies were enrolled in sequence, with seven consecutive batches constituting one cycle. The NDCM consisted of a quarantine nursery (qNur) facility in Quebec, Canada, followed by entry of the batch at ~40 days of age into a nearby late nursery and finisher facility of CDPQ that was initially seeded with multiple pathogens by bringing in growing-finishing pigs from multiple farms and with a known history of polymicrobial disease, as described by Putz et al. [16]. The disease challenge was maintained through a continuous flow system, in which each new batch was exposed via direct and indirect contact to pathogens present in the batch that was entered into the challenge facility three weeks earlier. At ~67 days of age, pigs were moved from the late nursery to the finisher, which shared the same air space. This design resulted in a high but variable disease pressure that reflects commercial production environments more closely than experimental infection models with specific pathogens.

### Performance and resilience phenotypes

The performance and resilience phenotype data for 3,285 pigs from the 50 batches of the NDCM that were analyzed by Cheng et al. [17] were available. All phenotypes were collected by trained research staff from CDPQ. Details on the collection and derivation on the performance and resilience traits were described by Putz et al. [16] and Cheng et al. [17].

In the context of the NDCM, we use the term “resilience” to denote an animal’s ability to maintain health and performance when exposed to a natural polymicrobial disease pressure. Because the NDCM represents a complex and dynamic exposure scenario in which multiple pathogens and disease processes co-occur and vary across time, pens, and batches, comprehensive and standardized quantification of pathogen burden (i.e., pathogen-specific loads across all relevant agents) and lesion scores across all animals was not feasible within the scope of this study. Accordingly, the phenotypes evaluated here should not be interpreted as pathogen-specific resistance. Instead, we interpret the recorded health and performance outcomes as practical proxy phenotypes of resilience/robustness under challenge.

Specifically, we consider multiple outcome domains. First, clinical health scores provide standardized assessments of observed clinical severity at defined time points, mortality captures the most severe health outcome, and antibiotic treatment counts reflect a combination of clinical need and management decisions (e.g., treatment thresholds) that are nevertheless closely linked to disease severity experienced under challenge. Within this domain, qNurHS1 (D5 post-entry into the quarantine nursery) reflects a combination of early-life adaptation to weaning, transport, environmental and diet change, and early health status. Thus, qNurHS1 is a possible early predictor of resilience, as it captures early robustness and coping capacity, which are components of resilience. Second, growth, feed intake, and efficiency traits measured during and after the challenge (ADG in the nursery and finisher, and feed intake and feed conversion ratio in the finisher) also capture resilience in the sense that more resilient animals are expected to better sustain growth and feed efficiency under variable disease pressure. We, therefore, consider these performance traits, particularly those recorded during the challenge nursery and finisher phases, as complementary indicators of resilience that integrate the cumulative impact of subclinical and clinical disease, immune activation, and recovery on productivity in a commercial-like environment with high disease pressure.

Based on this framework, traits were summarized by production phase to reflect both health-related outcomes and performance under challenge: the quarantine nursery (qNur), challenge nursery (cNur), and the finisher (Fin), as well as across the cNur and Fin phases (All). For the qNur phase, traits included average daily gain from 21 to 40 days of age (qNurADG) and health scores that were assigned to each pig by trained personnel using a standardized clinical scoring system (1 = severe clinical signs with wasting; 2 = severe clinical signs without wasting; 3 = mild to moderate clinical signs with or without wasting; 4 = mild clinical signs without wasting or light wasting without other clinical signs; 5 = perfect health status), shortly after arrival (qNurHS1, D5 post-entry) and at the end of the qNur phase (qNurHS2). In the cNur and Fin phases, recorded traits included growth rate (cNurADG, FinADG), feed intake and feed conversion ratio (FCR) in the finisher, health scores (cNurHS, FinHS), the number of individual antibiotic treatments received in cNur (cNurTRT), in Fin (FinTRT), and across cNur and Fin (AllTRT), and mortality in the corresponding phases (cNurMOR, FinMOR, and AllMOR). Carcass traits were obtained from the slaughter plant and included hot carcass weight (CWT), backfat depth (BFD), loin depth (CLD), dressing percentage (DRS), and lean yield (LYD). Details of all phenotypes recorded have been described by Putz et al. [16] and Cheng et al. [17].

### Genotyping and quality control

Genotyping of pigs was carried out using the Axiom Porcine 660 K genotyping array (Affymetrix, Santa Clara, CA, USA) at Delta Genomics (Edmonton, AB, Canada). Raw single-nucleotide polymorphism (SNP) intensities were processed by Delta Genomics with Axiom Analysis Suite, separately for each cycle, using the manufacturer’s default settings. After quality control (minor allele frequency > 0.05, SNP call rate > 0.90, and individual call rate > 0.90), genotypes for 435,172 autosomal SNPs on 3,205 pigs remained and were used to construct the genomic relationship matrix, consistent with earlier studies of this population [16, 17].

### Blood-omics data collection

Blood samples collected at ~27 days of age in the quarantine nursery from pigs in 15 batches, for a total of 836 pigs, were used to measure blood transcriptome, proteome, and metabolome profiles. Blood samples for RNA-seq were collected using Tempus Blood RNA Tubes (Thermo Fisher Scientific, USA) and processed for RNA-seq as described by Lim et al. [18]. Blood samples for proteomics and metabolomics were collected in K2 ethylenediaminetetraacetic acid (EDTA) tubes (BD Vacutainer^®^ Blood Collection Tubes, USA). The plasma was isolated by centrifugation at 3,000 r/min and 4 °C for 10 min, and the plasma layer (top) was aliquoted by transfer pipette into Thermo Scientific™ Nunc™ barcoded tubes. Samples were frozen at −80 °C immediately after processing for subsequent analyses.

The blood transcriptome was profiled using the QuantSeq 3′ mRNA-Seq Library Prep Kit FWD for Illumina with the RNA Removal Solution–Globin. Libraries were prepared from 500 ng of total blood RNA per pig, pooled and sequenced as single-end 50 bp reads in two lanes on an Illumina HiSeq 3000 system (Illumina, USA), and raw reads were processed (quality control, trimming and alignment to the *Sus scrofa* reference genome), as described by Lim et al. [18]. Gene counts were TMM-normalized in EdgeR [19], converted to counts per million (CPM), and log_2_-transformed as log_2_(CPM +1). Gene-wise mixed models that included RNA integrity number (RIN) [20] and entry age as covariates, sequencing batch as a fixed effect, and pen within batch as a random effect, were used to obtain residual expression values for downstream analyses. After filtering and preprocessing, RNA data on 15,872 genes were retained.

The plasma metabolome was quantified using proton nuclear magnetic resonance (NMR) spectroscopy at The Metabolomics Innovation Centre (TMIC) of the University of Alberta (AB, Canada; https://www.metabolomicscentre.ca/), following established sample preparation and spectral acquisition protocols, with details reported by Dervishi et al. [21]. Metabolites below the limit of detection in > 20% of samples or with ≥ 20% missing values were excluded, and the remaining missing values were imputed using metabolite-specific medians. Metabolite concentrations were log2-transformed and residualized using metabolite-wise mixed models with entry age as a covariate, batch as a fixed effect, and pen within batch as a random effect; resulting residuals were used for downstream analyses. After preprocessing, data on 44 metabolites were retained.

The plasma proteome was quantified using Thermo Scientific™ TMT™ 11-plex Tandem Mass Tag labelling kits and reagents, following the manufacturer’s protocol. Downstream peptide identification and protein summarization were performed, as described by Chen et al. [22]. The analytical runs that were used quantified partially overlapping sets of proteins and, therefore, the proportion of missing values increased when data from runs were combined. As shown in Fig. S1, the proportion of missingness for a protein was not related to its average abundance in reference samples, supporting the assumption that missing values were missing at random. We therefore used K-nearest neighbor (KNN) imputation to fill in missing data, which has been shown to perform well for missing-at-random proteomics data [23]. To validate the imputation, we randomly selected 100 proteins, set their observed values to missing, imputed them using KNN, and calculated the Pearson correlation between original and imputed values; this procedure was repeated 100 times. Fig. S2 shows boxplots of the resulting correlations by protein. Proteins with an average imputation accuracy greater than 0.4 were retained for downstream analyses, leaving data on 354 proteins.

### Computation of -omics relationship matrices

For the subset of 836 pigs with complete multi-omics data, similarity or relationship matrices among individuals were constructed based on each -omics data layer for use in phenotype prediction. The genomic relationship matrix ***G*** was built from SNP genotypes using the preGSf90 module of the BLUPF90 family [24], following the approach described by Cheng et al. [17]. Briefly, ***G*** was computed separately for pigs from each source PigGen Canada member company and then assembled into a block-diagonal matrix; off-diagonal blocks were set to zero to restrict relationships to within-company comparisons and to obtain pooled within-company variance components.

To compute similarity matrices for the non-genomic omics layers, we used adjusted and standardized feature values obtained during the omics preprocessing described above. For clarity, we summarize below the mixed-model framework used to obtain these adjusted values and to define the notation used in subsequent equations; this is not an additional preprocessing step beyond that described in the omics data section. The feature values used to construct the similarity matrix for a given molecular feature (RNA, protein, or metabolite) were the residuals from the following mixed linear model

1 $$\boldsymbol{y} = \boldsymbol{1}\mu + \boldsymbol{Xb} + \boldsymbol{Z}_{pen}\boldsymbol{p} + \boldsymbol{e}$$

where ${\boldsymbol{y}}$ is the vector of log-transformed expression or abundance across pigs, $\mu$ is the overall mean for that feature; ${\boldsymbol{b}}$ is the vector of fixed effects; ${\boldsymbol{p}}$ is the vector of random effects of pen in qNur (within batch) effects, with ${\boldsymbol{p}} \sim N(0,\boldsymbol{ }{\boldsymbol{I}}{\sigma }_{p}^{2})$; and ${\boldsymbol{e}}$ is the vector of residuals, with ${\boldsymbol{e}} \sim N(0, {\boldsymbol{I}}{\sigma }_{e}^{2})$. The design matrices ${\boldsymbol{X}}$ and ${{\boldsymbol{Z}}}_{pen}$ link the feature phenotype for each pig to fixed and pen effects, respectively. For gene expression, fixed effects included batch, enrichment-toy status, entry age, and RNA integrity number (RIN) (as in Lim et al. [18], the eWO model). For proteins, fixed effects included batch and entry age (as in Chen et al. [22]). For metabolites, fixed effects included batch and entry age (as in Dervishi et al. [21]).

For each omics layer, the residuals from model (1) were standardized by dividing by the feature-specific residual standard deviation, yielding $n\times p$ matrices ${{\boldsymbol{M}}}_{omics}$ of features with mean zero and unit variance (rows = pigs, columns = expressed genes, proteins, or metabolites). For each omics layer, the corresponding similarity matrix was then computed aswhich quantifies pairwise similarity among pigs based on that omics information source.

2 $$\boldsymbol{K}_{omics} = \frac{1}{p}\boldsymbol{M}_{omics}\boldsymbol{M}^{\prime}_{omics}$$

### Mixed linear prediction models

For each resilience trait, data from the 836 pigs with complete multi-omics profiles were analyzed using a linear mixed model to obtain BLUPs of phenotypes. In matrix form, the model can be written as

3 $$\boldsymbol{y} = \boldsymbol{1}\mu + \boldsymbol{Xb} + \boldsymbol{Z}_{pen}\boldsymbol{p} + \boldsymbol{Z}_{litter}\boldsymbol{l} + \sum\limits_{omics}\boldsymbol{Z}_{omics}\boldsymbol{u}_{omics} + \boldsymbol{e}$$

where ${\boldsymbol{y}}$ is the vector of observed phenotypes; $\mu$ is the overall mean; ${\boldsymbol{b}}$ is the vector of fixed effects, specified for each trait as in Cheng et al. [17]; ${\boldsymbol{p}}$ is the vector of random pen effects in qNur, with ${\boldsymbol{p}} \sim N(0, {\boldsymbol{I}}{\sigma }_{p}^{2})$; ${\boldsymbol{l}}$ is the vector of random common litter environmental effects, with ${\boldsymbol{l}} \sim N(0, {\boldsymbol{I}}{\sigma }_{l}^{2})$; and ${\boldsymbol{e}}$ is the vector of random residuals, with ${\boldsymbol{e}} \sim N(0, {\boldsymbol{I}}{\sigma }_{e}^{2})$. The term ${{\boldsymbol{u}}}_{omics}$ denotes the random animal effect associated with a given omics relationship matrix, where “omics” refers to genomic (G), transcriptomic (T), proteomic (P), or metabolomic (M) layers. For each omics layer, we assumed ${{\boldsymbol{u}}}_{omics} \sim N(0,{ {\boldsymbol{K}}}_{omics}{\sigma }_{omics}^{2})$, where ${{\boldsymbol{K}}}_{omics}$ is the corresponding relationship matrix (G, TRM, PRM, or MRM) and ${\sigma }_{omics}^{2}$ is its variance component. The design matrices ${\boldsymbol{X}}$, ${{\boldsymbol{Z}}}_{pen}$, ${{\boldsymbol{Z}}}_{litter}$, and ${{\boldsymbol{Z}}}_{omics}$ link records to fixed, pen, litter, and omics-based random animal effects, respectively.

To assess the contribution of omics information to prediction of each trait, model (3) was fitted with all possible combinations of one to four omics-based random animal effects with their corresponding omics relationship matrices. In the following, different combinations are denoted by the corresponding letters, i.e., G, T, P, or M for single-omics models and, e.g., GTP for the model that includes genomic, transcriptomic, and proteomic effects.

When two or more omics layers were used, we considered two approaches. In the first approach, a separate random animal effect ${{\boldsymbol{u}}}_{omics}$ was fitted for each omics matrix, each with its own variance component ${\sigma }_{omics}^{2}$, and effects associated with different omics layers were assumed to be uncorrelated. In the second approach, a combined relationship matrix was used and the term $\sum_{omics}{{\boldsymbol{Z}}}_{omics}{{\boldsymbol{u}}}_{omics}$ in model (3) was replaced by a single random animal effect ${{\boldsymbol{u}}}_{c} \sim N(0, {\boldsymbol{C}}{\sigma }_{c}^{2})$, where ${\boldsymbol{C}}$ is the combined relationship matrix constructed by the unweighted mean (i.e., equal-weight average) of corresponding omics-specific matrices as

4 $$\boldsymbol{C} = \frac{1}{n_{K}} \sum\limits^{n_{K}}_{k=1} \boldsymbol{K}_{k}$$

where ${{\boldsymbol{K}}}_{k}$ denotes one of the selected omics-specific matrices and ${n}_{K}$ is the number of omics layers included, and ${\sigma }_{c}^{2}$ is the corresponding variance component. This combined-kernel parameterization was used as a parsimonious alternative that reduces the number of variance components to be estimated, which can improve numerical stability for the sample size of our data. We note that using an unweighted mean implicitly assumes that each included omics layer is equally informative after scaling, which likely is not a valid assumption. This approach was included as a pragmatic aggregation or baseline, while trait-specific contributions of individual omics layers were assessed through the multi-kernel models in the first approach.

Model (3) was fitted to data on all 836 pigs using the ASReml 4.0 software [25] in order to estimate variance components. For each trait and model, the proportion of phenotypic variance explained by omics information was computed from the estimated variance components. For multi-omics models that fitted multiple random animal effects, the total proportion explained by the combined omics data was calculated as

5 $$\hat{h}^{2}_{omics.total} = \frac{\sum\nolimits_{k} \hat{\sigma}^{2}_{omics,k}}{\hat{\sigma}^{2}_{p}+\hat{\sigma}^{2}_{l} + \sum\nolimits_{k} \hat{\sigma}^{2}_{omics,k} + \hat{\sigma}^{2}_{e}}$$

where ${\widehat{\sigma }}_{p}^{2}$, ${\widehat{\sigma }}_{l}^{2}$, and ${\widehat{\sigma }}_{e}^{2}$ are the estimated variance components for pen, litter, and residual effects, respectively, and ${\widehat{\sigma }}_{omics,k}^{2}$ denotes the variance component for the $k$^th^ omics layer. The proportion contributed by each individual omics layer was obtained analogously by replacing the numerator with ${\widehat{\sigma }}_{omics,k}^{2}$.

### Training and validation strategy

Company and batch information for the 836 pigs with complete multi-omics data is summarized in Table 1. Prediction performance of the different models was assessed using a leave-one-batch-out cross-validation scheme within companies that had at least three batches (companies B, D, and E). For each validation round, one batch was designated as the validation set and all remaining batches from the same company were used as the training set. Model (4) was fitted to the training data, and random animal effects were predicted (BLUPs) for pigs in the validation batch based on their genomic and/or omics relationships to the animals in training. Observed phenotypes were pre-adjusted using a baseline mixed model with the same fixed effects as Model (4) and random pen and litter effects, but excluding any omics- or genomic-based animal effects. The resulting corrected phenotypes (residualized for fixed, pen, and litter effects) were used in all prediction evaluations. Prediction accuracy was calculated as the Pearson correlation between predicted phenotypes (sum of random animal effects) and adjusted phenotypes for pigs in the validation batch. For each trait and model, average prediction accuracy was obtained as the sample-size–weighted mean of the batch-specific correlations across all validation batches. Thus, resulting prediction accuracies reflect the ability to predict phenotype for a new batch from the same company under similar challenge conditions.

**Table 1:** Batch, cycle, and company information for the 836 pigs used for analysis

Cycle	Total number	Batch	Company	Number
Cycle 4	144	4D	A	67
		4E	E	34
		4G	D	43
Cycle 5	239	5A	E	64
		5B	A	68
		5F	B	53
		5G	F	54
Cycle 6	102	6C	D	49
		6E	B	53
Cycle 7	351	7A	E	60
		7C	C	45
		7D	B	67
		7E	D	54
		7F	F	54
		7H	B	71

Company indicates the source breeding company (PigGen Canada member breeding company) from which the batch originated

For binary mortality traits, we additionally evaluated discriminative performance using the area under the receiver operating characteristic curve (ROC AUC). In each leave-one-batch-out fold, out-of-sample prediction scores for pigs in the held-out batch were defined as the sum of the predicted random animal effects that were fitted from the training model. Because some validation batches contained only controls or only cases (resulting in batch-specific AUC to be undefined), we computed a pooled (overall) AUC by concatenating the out-of-sample predictions from all folds such that each pig contributed exactly once with an out-of-sample score.

## Results

### Summary statistics and genetic parameter estimates of the performance and disease resilience traits

Summary statistics and estimates of genetic parameters for the performance and disease resilience traits based on the 836 pigs used in this study are in Table 2. Estimates of genetic parameters were obtained from genomic animal models that included the same fixed effects as in Cheng et al. [17], a random animal effect based on the genomic relationship matrix ${\boldsymbol{G}}$, a random litter effect, and a residual term. Production and carcass traits showed moderate to high heritability estimates. Heritability estimates for ADG were moderately high for all phases (≈ 0.20 to 0.30), and other production traits such as ADFI, FCR, and RFI also showed moderate heritability estimates. Carcass traits had heritability estimates ranging from low–moderate to high (0.25 to 0.72). In contrast, health scores had low heritability estimates (0.01 to 0.09) in all phases, while antibiotic treatment counts (TRT) and mortality traits (MOR) generally had very low heritability, with estimates close to zero for FinTRT and FinMOR. Estimates of litter effects were small for most traits (< 0.06) but were moderate (0.10 to 0.17) for qNurADG, FinADG, FCR, CWT, and DRS, indicating some shared early-life environmental influences on these traits.

**Table 2:** Summary statistics and estimates of the proportion of phenotypic variance explained by genetics (heritability) and litter effects for the performance and disease resilience traits based on the 836 pigs analyzed

Trait	N	Mean	Standard deviation	Heritability (SE)	Litter (SE)
qNurADG	836	0.31	0.09	0.26 (0.10)	0.13 (0.05)
qNurHS1	836	4.84	0.36	0.09 (0.08)	0.00 (0.00)
qNurHS2	836	4.85	0.35	0.02 (0.08)	0.04 (0.05)
cNurADG	836	0.28	0.16	0.30 (0.08)	0.04 (0.04)
cNurHS	822	4.59	0.49	0.06 (0.08)	0.02 (0.04)
cNurTRT	829	1.02	1.07	0.18 (0.08)	0.00 (0.04)
cNurMOR	836	0.11	0.32	0.15 (0.08)	0.00 (0.00)
FinADG	647	0.90	0.13	0.20 (0.09)	0.12 (0.06)
FinHS	703	4.82	0.38	0.01 (0.07)	0.06 (0.05)
FinTRT	647	0.15	0.39	0.00 (0.00)	0.05 (0.05)
AllTRT	647	1.05	1.16	0.15 (0.09)	0.00 (0.00)
FinMOR	741	0.13	0.33	0.00 (0.00)	0.00 (0.00)
AllMOR	836	0.23	0.42	0.11 (0.07)	0.00 (0.00)
ADFI	647	2.23	0.32	0.37 (0.11)	0.05 (0.05)
FCR	647	2.61	0.19	0.37 (0.11)	0.18 (0.06)
RFI	647	0.04	0.12	0.68 (0.09)	0.00 (0.00)
CWT	603	91.30	9.79	0.25 (0.13)	0.17 (0.07)
DRS	601	77.57	2.07	0.30 (0.13)	0.10 (0.07)
LYD	566	61.32	1.70	0.71 (0.11)	0.02 (0.05)
CLD	568	59.33	5.99	0.43 (0.11)	0.00 (0.00)
CBF	568	16.91	3.77	0.72 (0.11)	0.03 (0.05)

Standard deviation: Phenotypic SD was calculated as the square root of the estimated phenotypic variance from the corresponding univariate mixed model with genomic relationships fitted to the 836 pigs. *qNur* Quarantine nursery phase, *cNur *Challenge nursery phase, *Fin* Challenge finisher phase, *All* Overall challenge phase, *ADG* Average daily gain (kg/d), *HS* Health score (4 = clinical signs observed; 5 = no clinical signs), *TRT* The number of individual parenteral antibiotic treatments during that phase (adjusted to the days), *MOR* Mortality (0 = survived; 1 = died), *ADFI* Average daily feed intake (kg/d), *FCR* Feed conversion ratio, *RFI* Residual feed intake (kg/d), *CWT* Carcass weight (kg), *CBF* Carcass back fat (mm), *CLD *Carcass loin depth (mm), *DRS* Dressing percentage (%), *LYD* Lean yield (kg)

### Variance partitions with different combinations of omics relationship matrices

**Fig. 2:**
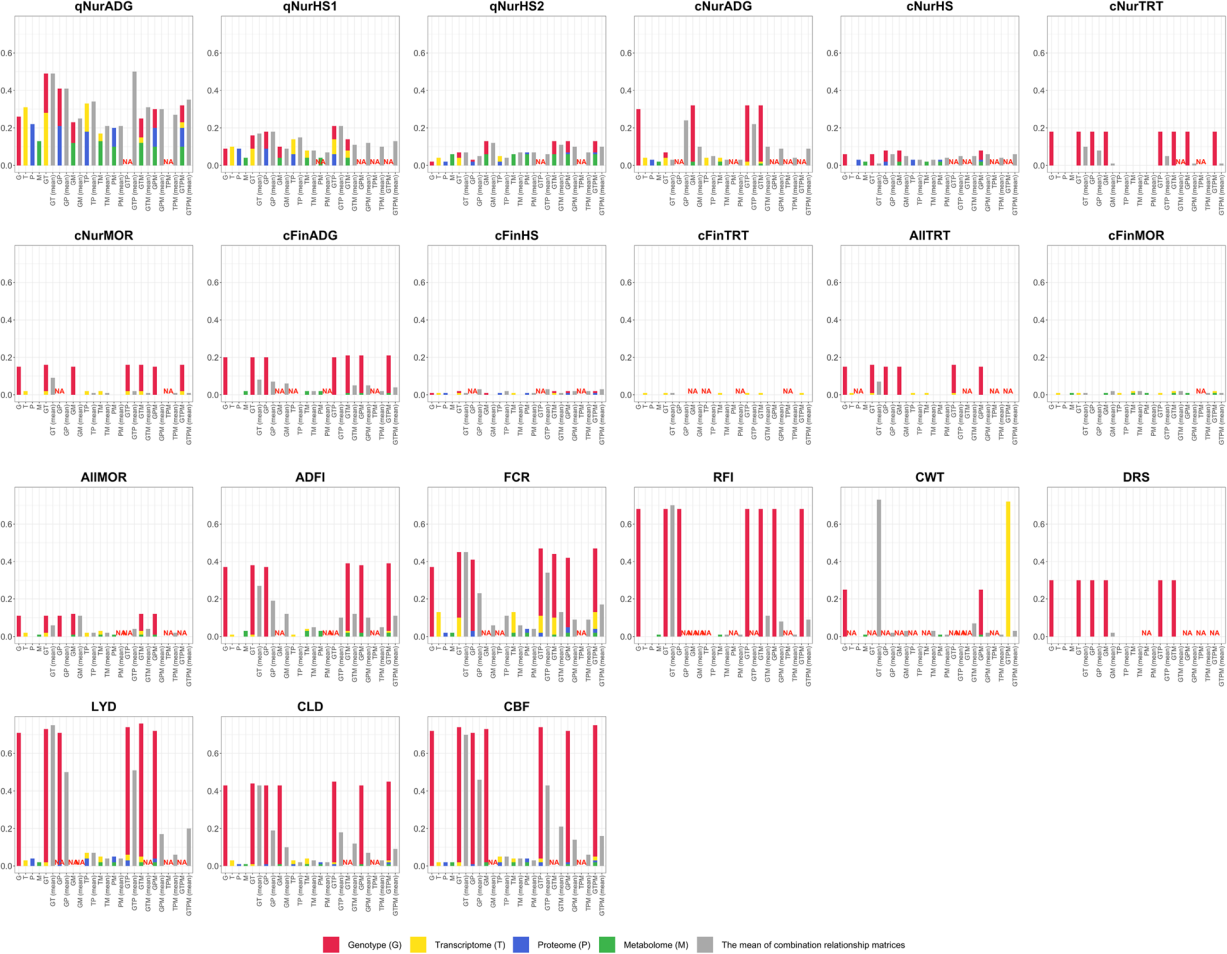
Estimates of the proportion of phenotypic variance explained by the different omics relationship matrices (genomics, G; transcriptomics, T; proteomics, P; metabolomics, M) for each performance and disease resilience trait, based on each omics data source independently or in combination. GT: G and T fitted jointly (two matrices), GT (mean): mean of G and T → one combined matrix, Grey bars refer to the use of a single combined relationship matrix across the omics layers used

Estimates of the proportion of phenotypic variance explained by the different similarity matrices and their combinations are shown in Fig. 2, with trait-specific estimates provided in Tables S1–S25. When each omics source was fitted separately (models G, T, P, M), model G generally explained a larger proportion of phenotypic variance than any single omics layer. Exceptions were mainly for traits measured in qNur, for which T explained slightly more variance than G, including for qNurADG, qNurHS1, and qNurHS2.

Adding a single omics layer to genomics (models GT, GP, GM, and their combined matrix versions) typically increased the proportion of variance explained (Fig. 2), with the largest gains observed for qNur traits and for FCR and CWT. For most other traits, increases in variance explained were small. Adding two or more omics layers without genomics (TP, TM, PM, TPM, and their combined matrix counterparts) resulted in very low proportions of variance explained, generally less than model G, even for the qNur traits, and these models were also more prone to not converge.

When two or more omics layers were added on top of genomics (e.g., models GTP, GPM, GTM, GTPM), the proportion of variance explained was higher than for model G, but gains were usually smaller than those obtained by adding only a single omics layer to genomics. Changes in variance explained were very similar whether omics layers were fitted as separate random effects or through a combined multi-omics relationship matrix (grey bars in Fig. 2), and genomics remained the dominant contributor to explained variance, with omics information providing useful additional signal only for a subset of traits.

### Phenotype prediction accuracy

**Fig. 3:**
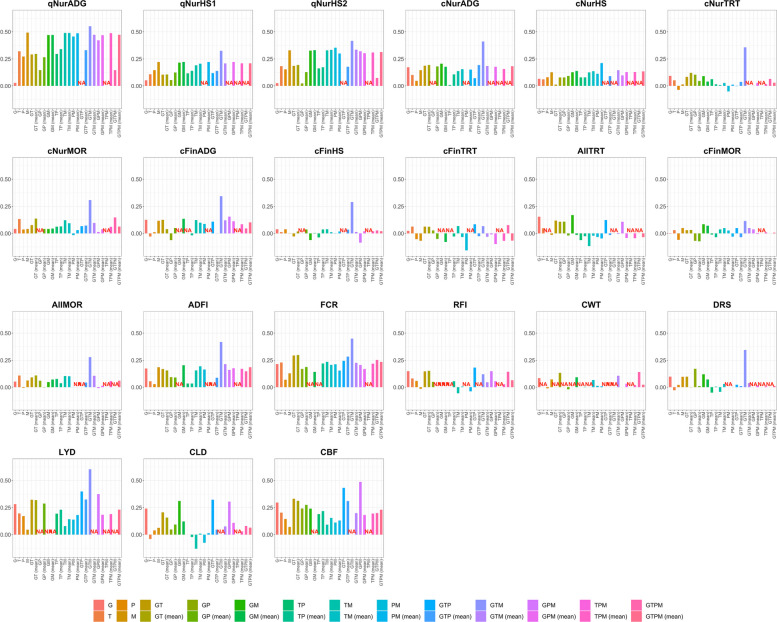
Estimates of the phenotype prediction accuracy based on 26 different combinations of omics relationship matrices (genomics, G; transcriptomics, T; proteomics, P; metabolomics, M) for each performance and disease resilience trait

Prediction accuracies for all trait and model combinations are summarized in Fig. 3, with numerical values in Table S26. Several of the most complex models failed to converge for specific traits, as indicated by NA in Table S26, and these were not interpreted further.

Accuracies from the genomic-only model (G) were low for most quarantine nursery and mortality traits and moderate for grow-finish performance and carcass traits. When a single omics source was used without genomics (T, P, or M), prediction performance was strongly trait-specific. For the three qNur traits, all single omics models substantially outperformed model G. For qNurADG, accuracies were 0.32, 0.27 and 0.49 for T, P, and M, compared with 0.03 for G. For qNurHS1 and qNurHS2, all three omics layers improved on G, with M reaching accuracies of 0.22 and 0.33, respectively. Single omics models also gave higher accuracies than G for several other resilience and performance traits, including for cNurHS (0.13 for M vs. 0.07 for G), cNurMOR (0.13 for T vs. 0.04 for G), AllMOR (0.11 for T vs. 0.05 for G), ADFI (0.19 for M vs. 0.17 for G), and FCR (0.23 for T vs. 0.21 for G). For many later grow-finish and carcass traits, however, accuracy from G was similar to or better than that of any other single omics model.

Models that combined two or more omics layers without genomics (TP, TM, PM, TPM, and their combined matrix versions) also showed trait dependent behavior in terms of accuracy. For some qNur traits, these multi-omics models performed similar to the best single omics model. For qNurADG, TM and $\overline{\mathrm{TM} }$ reached 0.49, very close to M (0.49), and PM and $\overline{\mathrm{PM} }$ achieved accuracies of 0.46 and 0.49. For qNurHS2, PM gave the highest accuracy among all models without genomics (0.35), and $\overline{\mathrm{TM} }$ had an accuracy of 0.33, both clearly above the accuracy for G (0.03). For cNurHS, $\overline{\mathrm{PM} }$ had the highest accuracy among the models that did not include G (0.21 vs. 0.13 for M and 0.07 for G). In contrast, for many traits recorded later in the grow-finish phase and for most carcass traits, TP, TM, PM, and TPM had accuracies close to zero or negative and did not improve on the best single omics model or on G.

Adding a single omics layer to genomics (GT, GP, GM, and their combined matrix versions) generally improved or, at worst, did not reduce prediction accuracy relative to G, although the size of the gain and the most informative omics layer depended on the trait. For qNurADG, accuracy increased from 0.03 for G to 0.29 for GT and 0.47 for GM. For qNurHS1 and qNurHS2, GT, GP, and GM all improved on G, with GM reaching accuracies of 0.22 and 0.33, respectively. For FCR, GT and $\overline{\mathrm{GT} }$ increased accuracy from 0.21 for G to 0.29 and 0.30, respectively, and for CWT, $\overline{\mathrm{GM} }$ improved accuracy from 0.09 for G to 0.14. GP provided the largest gain in accuracy among single omics layer additions to G for several traits, such as HHB, for which GP reached an accuracy of 0.17 compared to 0.10 for G. For other traits, including FinTRT and several carcass traits, adding any single omics layer to G changed accuracy very little and sometimes reduced it, and no single addition showed a clear advantage over G.

When two or more omics layers were added to G (GTP, GTM, GPM, GTMP, and their combined matrix versions), prediction accuracies increased further for many traits. The combination of G, T, and M (GTM or $\overline{\mathrm{GTM} }$) most frequently achieved the highest or near highest accuracies. For example, GTM and $\overline{\mathrm{GTM} }$ reached an accuracy of 0.55 for qNurADG, of 0.33 and 0.42 for, respectively, qNurHS1 and qNurHS2, 0.36 for cNurTRT, 0.31 for cNurMOR, 0.29 for FinHS, 0.28 for AllMOR, 0.42 for ADFI, and 0.45 for FCR. For some traits other multi-omics combinations performed best, such as GTP for CLD and $\overline{\mathrm{GPM} }$ for CBF. Adding a fourth omics layer (GTMP or $\overline{\mathrm{GTMP} }$) did not consistently improve accuracy and in some cases reduced performance compared to the use of G and one or two other omics layers.

For mortality traits, we additionally evaluated discrimination performance using AUC (Table S27). Overall, AUC values were modest but consistently above random for most models, with genomics-only AUC ranging from 0.48 to 0.56 across phases. The best-performing multi-omics combinations achieved higher AUCs, most notably the GTM model, which yielded the highest AUC for cNurMOR (0.66) and AllMOR (0.65), and a moderate AUC for cFinMOR (0.56). These results complement the correlation-based accuracies in Table S27 and support the conclusion that integrating transcriptomic and metabolomic information can improve discrimination for mortality outcomes under the disease challenge.

In summary, prediction accuracies from models using combined multi omics relationship matrices typically closely mirrored those from models that fitted each omics matrix separately, with no clear overall benefit of either approach.

## Discussion

This study evaluated whether blood-based multi-omics profiles of young and clinically healthy pigs can improve prediction of performance and disease resilience traits of pigs under a natural polymicrobial challenge. Using genomic, transcriptomic, proteomic, and metabolomic data collected from blood of clinically healthy pigs in a high health quarantine nursery, we compared BLUP models based on single and multiple omics layers, with or without genomics, for traits measured prior to, during, and after a natural polymicrobial disease challenge. Three main conclusions emerged. First, genomics remained the strongest single information source for most traits, especially for later grow-finish performance and carcass traits under disease, but phenotype prediction accuracies for health, treatment, and mortality traits were low when using genomics alone, as expected based on the low heritability of these traits. Second, early life metabolomic and transcriptomic profiles contained substantial predictive signal for traits prior to the challenge, and for mortality and some feed efficiency traits under challenge, in several cases matching or exceeding the accuracy of genomic predictions. Third, combining omics layers with genomics improved prediction accuracy for many traits, with the genomic—transcriptomic—metabolomic combination (GTM) most often among the best performing models, while also including proteomics did not always improve prediction accuracies.

These findings align with and extend previous work on prediction of resilience and robustness in livestock. Earlier studies in pigs and other species have shown that genomic prediction can capture genetic variation in survival, health scores, and health treatment records, but with lower accuracies than for classical production traits because of their low heritability, heterogeneous pathogen exposure, and coarse phenotyping of disease outcomes [4, 9, 16]. Our genomic heritability and accuracy estimates are consistent with this pattern and reinforce the view that genomics alone is often insufficient for precise prediction of phenotypes and breeding values for disease resilience. At the same time, studies that integrated metabolomic or transcriptomic data with genomic information in livestock and crops have generally reported small but sometimes meaningful increases in phenotype prediction accuracies for growth, carcass, and other complex traits, but with considerable heterogeneity across traits and data sets [26–29]. The modest improvements we observed for many traits, and the clearer gains for a subset of resilience and feed efficiency traits, are consistent with this broader literature.

To better understand why the value of omics differed by trait and why adding more omics layers did not necessarily improve prediction accuracies, we considered the variance partitions from models fit with different combinations of relationship matrices. Because these relationship matrices were constructed from molecular profiles that were measured once in clinically healthy pigs, the variance attributed to an omics kernel is best interpreted as predictive covariance captured by molecular similarity at the time of sampling, which can reflect a blend of genetic signal, early-life environment, and transient physiology, rather than the strictly additive genetic variance that is expected to be captured by genomic relationship matrices. This framing helps explain the stage-specific utility of omics: early-life transcriptomic and metabolomic states may capture upstream biology (e.g., immune readiness and metabolic status) that later influences susceptibility and response under challenge, whereas later grow-finish and carcass outcomes remain more strongly tied to the genetic relationships captured by genomics. The variance partitions also suggest that different omics layers can carry overlapping information, such that adding additional correlated kernels can lead to diminishing returns and, in finite samples, create identifiability challenges when kernels compete to explain the same underlying signal. Collectively, these considerations support viewing different omics layers as complementary predictors for phenotype or risk stratification and management, while genomics remains the appropriate backbone for breeding-oriented selection.

Importantly, because pathogen burden and lesion scores were not measured in this natural, polymicrobial disease challenge, improved prediction from using blood omics data should not be interpreted as evidence of pathogen-specific resistance. Rather, the added signal from transcriptomic and/or metabolomic similarity likely reflects early-life physiological and immune states, shaped by both genetic predisposition and recent environmental exposures, that are predictive of subsequent health and productivity under variable disease pressure. In this sense, the most immediate practical value of pre-challenge blood multi-omics data is to enhance early phenotype or risk prediction to support targeted management and intervention strategies, while genomics remains central for breeding value prediction and selection.

Consistent with this interpretation, pre-challenge blood-based omics profiles from clinically healthy young pigs provided complementary information to genomics in several important ways. When used as single sources, transcriptomic, proteomic, or metabolomic models achieved phenotype prediction accuracies that were comparable to or greater than those from genomics for roughly half of the traits. This was most evident for growth prior to challenge, and for health traits and for some mortality and feed efficiency traits under challenge, where metabolomic and, to a lesser extent transcriptomic models, performed particularly well. These patterns suggest that early life omics profiles capture information on the physiological and immune status of the animal that reflects both genetic predisposition and recent environmental influences, and that this information is predictive of how pigs respond to the disease challenge in the nursery. In contrast, for many finisher growth and carcass traits, omics-only models provided limited benefit beyond genomics, likely because early-life blood-omics profiles capture short-term physiological status but do not fully reflect the longer-term environmental and management factors that shape these traits.

Models that combined two or more omics layers without genomics (TP, TM, PM, and TPM, including their combined matrix versions) also showed trait-specific behavior. For some early phase traits, such as growth prior to the challenge, and for health scores, these multi-omics models performed similarly to the best single omics model, and in a few cases, combinations such as proteome plus metabolome (PM) gave the highest accuracies among all models that did not include genomics. However, for many later performance and carcass traits, these models produced low or unstable accuracies and did not improve on genomics or on the best single omics source. Given the limited sample size, adding multiple correlated omics layers without the anchoring effect of genomic relationships likely increases model complexity more than the information contained in these additional data. In practice, omics-only models may be useful when genotyping is not feasible, but they do not appear robust enough to replace genomics across traits.

Combining omics layers with genomics was consistently more beneficial, but the optimal combination depended on the trait. Adding a single omics layer to genomics (GT, GP, or GM) typically improved or, at worst, did not reduce prediction accuracies, although the size of the gain and the most informative addition varied. For early growth, mortality, and feed efficiency traits, adding metabolomic or transcriptomic data often yielded clear improvements, while proteomic information provided the largest incremental gain for other traits. For several carcass traits and some health traits, however, none of the single omics additions gave a clear advantage over genomics and accuracies remained modest. When two or more omics layers were added on top of genomics, prediction accuracy increased further for many traits, with GTM and related models most frequently achieving the highest or near highest accuracies. This suggests that transcriptomic and metabolomic information capture partially distinct aspects of early life biology that are both relevant for later resilience and performance. At the same time, models that included all four omics layers (GTMP) did not systematically outperform simpler omics combinations and sometimes reduced accuracy, likely due to overparameterization and the inclusion of less informative or noisier omics data. These patterns are consistent with theoretical and empirical work indicating that, in finite samples, adding predictors with a low signal to noise ratio can dilute the contribution of more informative features [26, 29].

Our findings are partly consistent with, but also differ from, previous multi-omics prediction studies in other species. Xu et al. [27] combined genomic, transcriptomic, and metabolomic information for maize in a mixed model framework similar to ours and reported little or no improvement in prediction over genomics alone, whereas Guo et al. [26] found that integrating transcriptomic and metabolomic data with genomic information in maize substantially increased predictive ability compared with using genomics only. Evidence from other model systems has also been mixed, with reported benefits of adding transcriptomic or metabolomic information varying across traits and study designs [28, 29]. These heterogeneous findings across studies likely reflect differences in trait architectures, tissues and timing of sampling, health status at sampling, omics platforms, and the metric used to quantify prediction performance [30]. Together with our results, these studies illustrate that the benefit of multi-omics integration is highly context dependent and can vary with species, traits, tissues, omics platforms, and the definition of prediction accuracy.

We also compared models that fitted each omics layer using separate random effects with those using a single combined multi-omics relationship matrix. For most traits, the total proportion of variance explained and prediction accuracies were very similar between these two approaches, with differences typically small and no consistent advantage of either parameterization. This suggests that, under the current design and sample size, prediction is driven more by whether informative omics layers are included than by fine-scale attribution of variance to individual layers. From a practical standpoint, the combined multi-omics relationship matrix offers a parsimonious alternative that reduces the number of variance components to be estimated and can therefore improve numerical stability. Consistent with this, the most highly parameterized multi-kernel models, especially those combining multiple omics layers without genomics, were more prone to convergence problems. Together, these results highlight the need to balance model complexity against numerical stability when fitting high-dimensional multi-omics models for prediction applications.

Several limitations should be considered. First, the number of pigs with complete multi-omics profiles was modest relative to typical reference populations used for genomic prediction, which constrains the maximum achievable accuracy, especially for low heritability traits and for complex models. Second, the omics data were collected on blood at a single early time point, so we could not assess whether repeated measures or later sampling would further improve prediction accuracies. Third, the combined relationship matrix approach used an unweighted mean of the available omics relationship matrices, which is a parsimonious parameterization. In our data, prediction accuracies from the combined-matrix models were generally very similar to those from the corresponding multi-kernel models, suggesting that the equal-weight approximation is reasonable under the current design and sample size. Nevertheless, trait-specific weighting schemes (e.g., data-driven weights) may yield additional gains in some settings and should be evaluated in larger and independent populations. Fourth, all analyses were carried out within a single natural disease challenge system and, thus, the extent to which the predictive patterns observed here generalize to other genetic backgrounds, management systems, or pathogen spectra is not clear. Fifth, we did not perform external validation in an independent natural-challenge population; therefore, the stability of the relative ranking of omics layers and model combinations, and the magnitude of observed gains, should be interpreted as specific to the current NDCM design and reference set. Finally, we focused on linear mixed models with relationship matrices derived from standardized omics features. Alternative approaches, such as feature selection, sparse regression or non-linear methods, may extract different information from the same data and deserve investigation in future work.

Despite these limitations, our results have clear implications for improving disease resilience. For breeding programs, genomic prediction remains the appropriate route to estimate breeding values and selection, whereas pre-challenge blood multi-omics profiles collected on young and clinically healthy pigs can be leveraged as complementary biomarkers for management to improve early identification of at-risk animals and optimize interventions under commercial-like disease pressure. Translating these findings into practice will likely require reduced biomarker panels derived from transcriptomic and metabolomic profiles, cost–benefit evaluation by trait, and validation in independent populations and production environments. In addition, future work should prioritize external validation of the proposed multi-omics prediction patterns and model rankings in independent populations and production environments before practical deployment, and should evaluate data-driven weighting schemes for constructing a single combined kernel C (as an alternative to the equal-weight mean used here), assess whether repeated or later-time-point sampling improves predictive performance for downstream finisher and carcass traits, and incorporate more standardized and granular health phenotyping where feasible to better disentangle resilience components. While recent work has proposed integrating intermediate omics features into formal genetic evaluation frameworks for breeding value prediction, our focus here was on phenotype or risk prediction under disease challenge conditions and we therefore did not pursue multi-omics modeling for breeding value prediction [31, 32]. Future studies should also explore how multi-omics prediction can be integrated with improved phenotyping of resilience and with genetic evaluation models that explicitly account for heterogeneous environments and pathogen exposure.

## Conclusions

This study shows that blood-based multi-omics profiles collected on clinically healthy nursery pigs can enhance prediction of performance and disease resilience traits of pigs under a natural polymicrobial disease challenge. Genomic information remained the core predictor across traits, but early-life transcriptomic and metabolomic profiles, and their integration with genomics, provided meaningful gains in prediction accuracies for early growth and for health, mortality, and for some feed efficiency traits under disease, while adding all available omics layers did not systematically improve accuracies. These findings demonstrate that multi-omics data collected on young healthy pigs can capture biologically relevant variation in resilience before pathogen exposure and can be strategically combined with genomics to improve management and selection decisions. More broadly, this work provides a proof of concept for incorporating targeted multi-omics information into pig production and breeding programs to enhance robustness and health under disease challenges that are inherent to many commercial herds.

## Supplementary Information

Additional file 1: Fig. S1. Distributions of performance and resilience phenotypes in the natural disease challenge model (NDCM). Fig. S2. Plot of the log2 of average protein abundance for each protein against the percent missing based on the reference data. Fig. S3. Boxplot of Pearson correlation coefficients between the original and imputed values by proteins. Table S1. Summary statistics for the performance and disease resilience traits based on the 836 pigs analyzed. Table S2. Estimates of the proportion of phenotypic variance explained by omics relationship matrices and litter effects for the transcriptome BLUP model. Table S3. Estimates of the proportion of phenotypic variance explained by omics relationship matrices and litter effects for the proteome BLUP model. Table S4. Estimates of the proportion of phenotypic variance explained by omics relationship matrices and litter effects for the metabolome BLUP model. Table S5. Estimates of the proportion of phenotypic variance explained by omics relationship matrices and litter effects for the GTBLUP model. Table S6. Estimates of the proportion of phenotypic variance explained by omics relationship matrices and litter effects for the GTBLUP mean model. Table S7. Estimates of the proportion of phenotypic variance explained by omics relationship matrices and litter effects for the GPBLUP model. Table S8. Estimates of the proportion of phenotypic variance explained by omics relationship matrices and litter effects for the GPBLUP (mean) model. Table S9. Estimates of the proportion of phenotypic variance explained by omics relationship matrices and litter effects for the GMBLUP model. Table S10. Estimates of the proportion of phenotypic variance explained by omics relationship matrices and litter effects for the GMBLUP (mean) model. Table S11. Estimates of the proportion of phenotypic variance explained by omics relationship matrices and litter effects for the TPBLUP model. Table S12. Estimates of the proportion of phenotypic variance explained by omics relationship matrices and litter effects for the TPBLUP (mean) model. Table S13. Estimates of the proportion of phenotypic variance explained by omics relationship matrices and litter effects for the TMBLUP model. Table S14. Estimates of the proportion of phenotypic variance explained by omics relationship matrices and litter effects for the TMBLUP (mean) model. Table S15. Estimates of the proportion of phenotypic variance explained by omics relationship matrices and litter effects for the PMBLUP model. Table S16. Estimates of the proportion of phenotypic variance explained by omics relationship matrices and litter effects for the PMBLUP (mean) model. Table S17. Estimates of the proportion of phenotypic variance explained by omics relationship matrices and litter effects for the GTPBLUP model. Table S18. Estimates of the proportion of phenotypic variance explained by omics relationship matrices and litter effects for the GTPBLUP (mean) model. Table S19. Estimates of the proportion of phenotypic variance explained by omics relationship matrices and litter effects for the GTMBLUP model. Table S20. Estimates of the proportion of phenotypic variance explained by omics relationship matrices and litter effects for the GTMBLUP (mean) model. Table S21. Estimates of the proportion of phenotypic variance explained by omics relationship matrices and litter effects for the GPMBLUP model. Table S22. Estimates of the proportion of phenotypic variance explained by omics relationship matrices and litter effects for the GPMBLUP (mean) model. Table S23. Estimates of the proportion of phenotypic variance explained by omics relationship matrices and litter effects for the TPMBLUP (mean) model. Table S24. Estimates of the proportion of phenotypic variance explained by omics relationship matrices and litter effects for the GTPMBLUP model. Table S25. Estimates of the proportion of phenotypic variance explained by omics relationship matrices and litter effects for the GTPMBLUP (mean) model. Table S26. Estimates of the phenotypic prediction accuracy based on 26 different combination of different omics relationship matrices (genomics, G; transcriptomics, T; proteomics, P; metabolomics, M) for each performance and disease resilience trait. Table S27. Estimates of the AUC for mortality prediction using 26 combinations of omics relationship matrices (genomics, G; transcriptomics, T; proteomics, P; metabolomics, M).

## Data Availability

The data analyzed in this study were obtained on commercial animals provided by members of the PigGen Canada consortium. Data can be made available upon reasonable request submitted to the corresponding author.

## Abbreviations

ADFI: Average daily feed intake (finisher phase)
AllMOR: Mortality collected during the entire challenge (as defined for cNurMOR)
AllTRT: Number of individual parenteral antibiotic treatments during the entire challenge (adjusted to 180 d)
BGAM: Bayesian generalized additive model
BLUP: Best linear unbiased prediction
CBF: Carcass back fat
CDPQ: The quebec provincial centre for population development
CLD: Carcass loin depth
cNur: Challenge nursery phase
cNurADG: Average daily gain measured during the challenge nursery phase
cNurHS: Health score assigned by trained people two weeks after entry into the challenge nursery phase
cNurMOR: Mortality collected during the challenge nursery phase (0/1; 0 = survived to slaughter; 1 = died before slaughter)
cNurTRT: Number of individual parenteral antibiotic treatments during the challenge nursery phase (adjusted to 27 d)
CWT: Carcass weight
DRS: Dressing percentage
EDTA: Ethylenediaminetetraacetic acid
eQTL: Expression quantitative trait loci
FCR: Feed conversion ratio
Fin: Finisher phase
FinADG: Average daily gain measured during the finisher phase
FinHS: Health score assigned by trained people six weeks after entry into the finisher phase
FinMOR: Mortality collected during the finisher phase (as defined for cNurMOR)
FinTRT: Number of individual parenteral antibiotic treatments during the finisher phase (adjusted to 100 d)
GP: Genomic prediction
GRM|G: Genomic relationship matrix
GWAS: Genome-wide association studies
KNN: K-nearest neighbor method
LYD: Lean yield
mQTL: Metabolite quantitative trait loci
MRM|M: Metabolomic relationship matrix
NDCM: Natural disease challenge model
pQTL: Protein quantitative trait loci
PRM|P: Proteomic relationship matrix
PRRSV: Porcine reproductive and respiratory syndrome virus
qNur: Quarantine nursery phase
qNurADG: Average daily gain measured during the quarantine nursery phase
qNurHS1: Health score assigned by trained people at day 5 post entry into the quarantine nursery phase
qNurHS2: Health score assigned by trained people at the end of quarantine nursery phase
RFI: Residual feed intake
SNP: Single nucleotide polymorphism
TMT: Tandem mass tag
TRM|T: Transcriptomic relationship matrix
