# Supplementary material for: Prediction of disease resilience of pigs using multi-omics data
Source: J Anim Sci Biotechnol. 2026 Jun 2;17:107. doi: 10.1186/s40104-026-01416-9 (PMC13227672; doi:10.1186/s40104-026-01416-9)
Supplement: Supplementary file 1 — Additional file 1: Fig. S1. Distributions of performance and resilience phenotypes in the natural disease challenge model (NDCM). Fig. S2. Plot of the log2 of average protein abundance for each protein against the percent missing based on the reference data. Fig. S3. Boxplot of Pearson correlation coefficients between the original and imputed values by proteins. Table S1. Summary statistics for the performance and disease resilience traits based on the 836 pigs analyzed. Table S2. Estimates of the proportion of phenotypic variance explained by omics relationship matrices and litter effects for the transcriptome BLUP model. Table S3. Estimates of the proportion of phenotypic variance explained by omics relationship matrices and litter effects for the proteome BLUP model. Table S4. Estimates of the proportion of phenotypic variance explained by omics relationship matrices and litter effects for the metabolome BLUP model. Table S5. Estimates of the proportion of phenotypic variance explained by omics relationship matrices and litter effects for the GTBLUP model. Table S6. Estimates of the proportion of phenotypic variance explained by omics relationship matrices and litter effects for the GTBLUP mean model. Table S7. Estimates of the proportion of phenotypic variance explained by omics relationship matrices and litter effects for the GPBLUP model. Table S8. Estimates of the proportion of phenotypic variance explained by omics relationship matrices and litter effects for the GPBLUP (mean) model. Table S9. Estimates of the proportion of phenotypic variance explained by omics relationship matrices and litter effects for the GMBLUP model. Table S10. Estimates of the proportion of phenotypic variance explained by omics relationship matrices and litter effects for the GMBLUP (mean) model. Table S11. Estimates of the proportion of phenotypic variance explained by omics relationship matrices and litter effects for the TPBLUP model. Table S12. Estimates of the proportion of phe [file 40104_2026_1416_MOESM1_ESM.docx]

**Supplementary Figures**

Supplemental Figure 1. Distributions of performance and resilience phenotypes in the natural disease challenge model (NDCM). (A) Histograms of continuous traits, including growth (qNurADG, cNurADG, FinADG), feed intake and efficiency (ADFI, FCR, RFI), treatment counts (cNurTRT, FinTRT, AllTRT), and carcass traits (CWT, DRS, LYD, CLD, CBF). (B) Frequency distributions of categorical traits, including mortality (cNurMOR, FinMOR, AllMOR) and clinical health scores (qNurHS1, qNurHS2, cNurHS, FinHS). Counts are based on non-missing observations.

A.

**
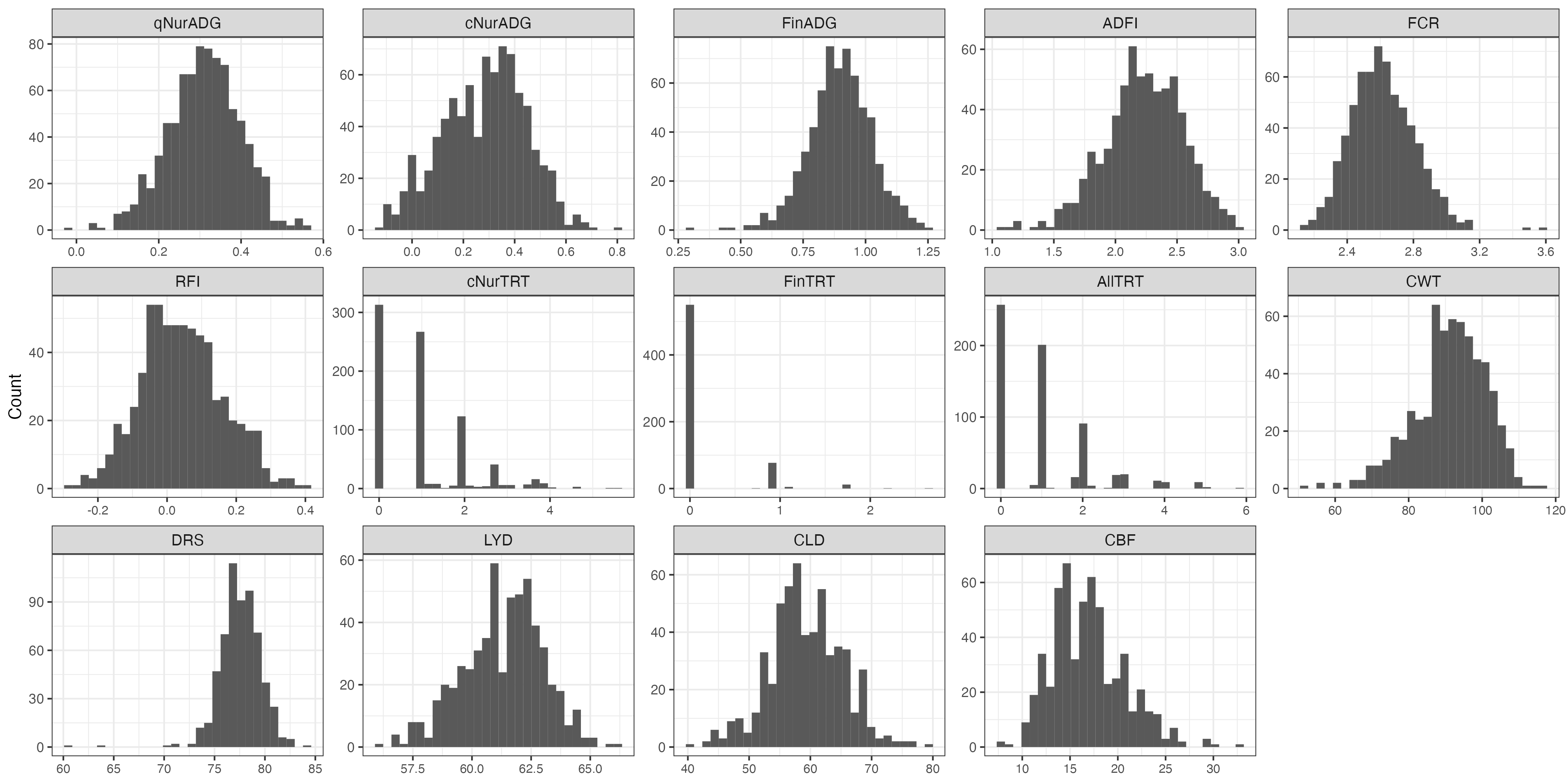
**

B.

**
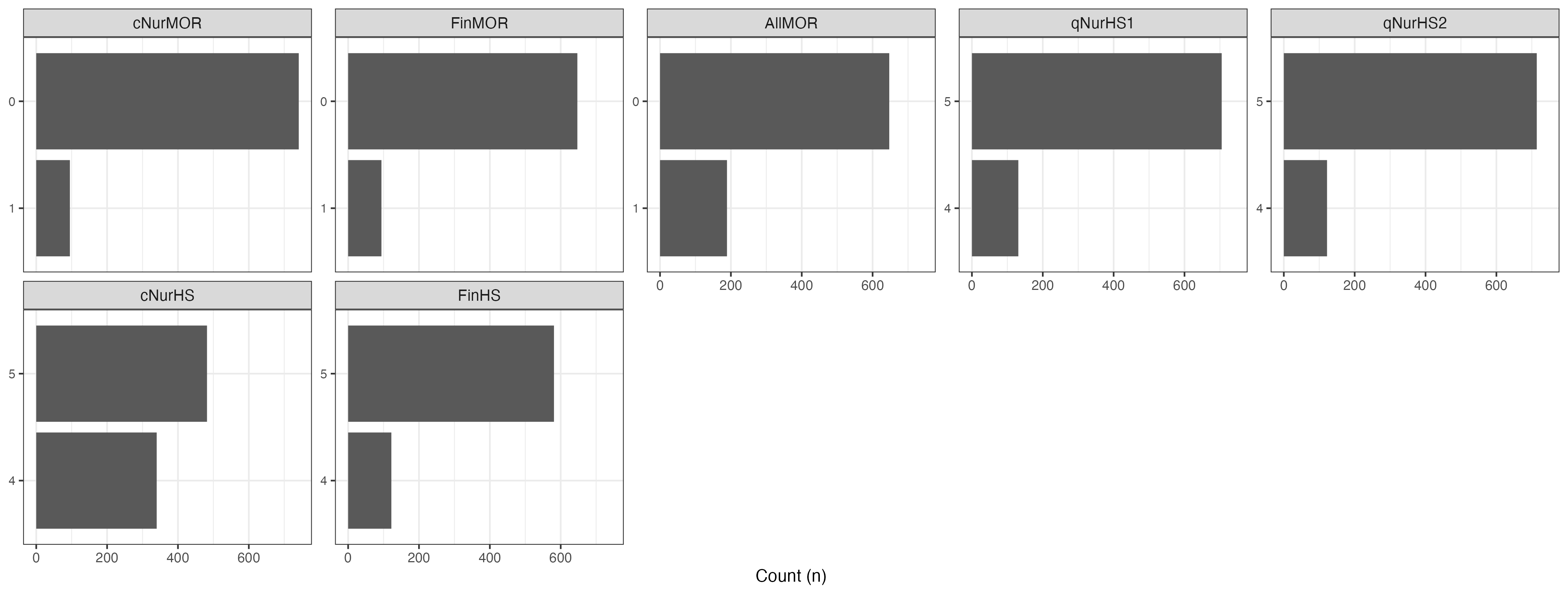
**

Supplemental Figure 2. Plot of the log2 of average protein abundance for each protein against the percent missing based on the reference data.


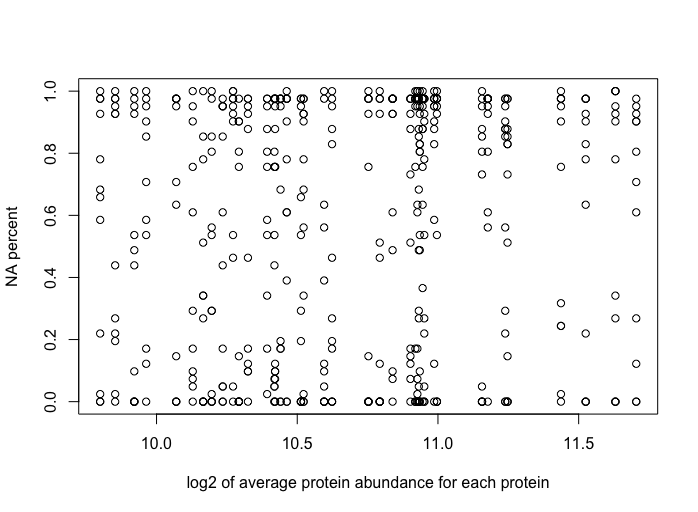


Supplemental Figure 3. Boxplot of Pearson correlation coefficients between the original and imputed values by proteins.

**Supplemental Tables**

Supplemental Table 1. Summary statistics for the performance and disease resilience traits based on the 836 pigs analyzed.

| **Trait** | **Level / Statistic** | **Overall** | **Missing** |
| --- | --- | --- | --- |
| qNurADG | Min-Max | -0.022 – 0.559 | 0 |
| qNurHS1 | 4 | 131 (15.67%) | - |
|  | 5 | 705 (84.33%) | - |
| qNurHS2 | 4 | 122 (14.59%) | - |
|  | 5 | 714 (85.41%) | - |
| cNurADG | Min-Max | -0.119 – 0.559 | 0 |
| cNurHS | 4 | 340 (41.36%) | - |
|  | 5 | 482 (58.64%) | - |
| cNurTRT | Min-Max | 0 – 5.59 | 7 |
| cNurMOR | 0 | 741 (88.64%) | - |
|  | 1 | 95 (11.36%) | - |
| FinADG | Min-Max | 0.294 – 1.251 | 189 |
| FinHS | 4 | 122 (17.35%) | - |
|  | 5 | 581 (82.65%) | - |
| FinTRT | Min-Max | 0 – 2.65 | 189 |
| AllTRT | Min-Max | 0 – 5.83 | 189 |
| FinMOR | 0 | 647 (87.31%) | - |
|  | 1 | 94 (12.69%) | - |
| AllMOR | 0 | 647 (77.39%) | - |
|  | 1 | 94 (12.69%) | - |
| ADFI | Min-Max | 1.071 – 3.011 | 189 |
| FCR | Min-Max | 2.123 – 3.566 | 189 |
| RFI | Min-Max | -0.288 – 0.403 | 189 |
| CWT | Min-Max | 52.1 – 117.1 | 233 |
| DRS | Min-Max | 60.30 – 84.00 | 235 |
| LYD | Min-Max | 55.90 – 66.00 | 270 |
| CLD | Min-Max | 41.00 – 80.00 | 268 |
| CBF | Min-Max | 7.50 – 32.50 | 268 |

qNur: Quarantine nursery phase; cNur: Challenge nursery phase; Fin: Challenge finisher phase; All: Overall challenge phase; ADG: Average daily gain (kg/day); HS: Health score (4 = clinical signs observed; 5 = no clinical signs); TRT: The number of individual parenteral antibiotic treatments during that phase (adjusted to the days); MOR: Mortality (0 = survived; 1 = died); ADFI: Average daily feed intake (kg/day); FCR: Feed conversion ratio; RFI: Residual feed intake (kg/day); CWT: Carcass weight (kg); CBF: Carcass back fat (mm); CLD: Carcass loin depth (mm); DRS: Dressing percentage (%); LYD: Lean yield (kg);

Supplemental Table 2. Estimates of the proportion of phenotypic variance explained by omics relationship matrices ($\hat{h}^{2}$) and litter effects ($c^{2}$) for the transcriptome BLUP model.

| **Trait** | **converge** | **h^2^_T_ (SE)** | **c^2^ (SE)** |
| --- | --- | --- | --- |
| qNurADG | conv | 0.31 (0.10) | 0.16 (0.04) |
| qNurHS1 | conv | 0.10 (0.06) | 0.01 (0.04) |
| qNurHS2 | conv | 0.04 (0.03) | 0.05 (0.05) |
| cNurADG | conv | 0.04 (0.04) | 0.11 (0.04) |
| cNurHS | conv | 0.00 (0.00) | 0.03 (0.04) |
| cNurTRT | conv | 0.00 (0.00) | 0.04 (0.04) |
| cNurMOR | conv | 0.02 (0.03) | 0.00 (0.00) |
| cFinADG | conv | 0.00 (0.00) | 0.18 (0.05) |
| cFinHS | conv | 0.01 (0.03) | 0.06 (0.05) |
| cFinTRT | conv | 0.01 (0.02) | 0.05 (0.05) |
| AllTRT | conv | 0.01 (0.02) | 0.03 (0.05) |
| cFinMOR | conv | 0.01 (0.02) | 0.00 (0.00) |
| AllMOR | conv | 0.02 (0.02) | 0.00 (0.00) |
| ADFI | conv | 0.01 (0.02) | 0.16 (0.05) |
| FCR | conv | 0.13 (0.08) | 0.26 (0.05) |
| RFI | conv | 0.00 (0.00) | 0.13 (0.05) |
| CWT | Not conv |  |  |
| DRS | conv | 0.00 (0.00) | 0.21 (0.05) |
| LYD | conv | 0.03 (0.03) | 0.27 (0.05) |
| CLD | conv | 0.03 (0.04) | 0.13 (0.06) |
| CBF | conv | 0.02 (0.03) | 0.27 (0.05) |

Supplemental Table 3. Estimates of the proportion of phenotypic variance explained by omics relationship matrices ($\hat{h}^{2}$) and litter effects ($c^{2}$) for the proteome BLUP model.

| **Trait** | **converge** | **h^2^_P_ (SE)** | **c^2^ (SE)** |
| --- | --- | --- | --- |
| qNurADG | conv | 0.22 (0.06) | 0.17 (0.04) |
| qNurHS1 | conv | 0.09 (0.05) | 0.02 (0.04) |
| qNurHS2 | conv | 0.02 (0.02) | 0.04 (0.05) |
| cNurADG | conv | 0.03 (0.03) | 0.11 (0.04) |
| cNurHS | conv | 0.03 (0.02) | 0.03 (0.04) |
| cNurTRT | conv | 0.00 (0.01) | 0.04 (0.04) |
| cNurMOR | conv | 0.00 (0.01) | 0.00 (0.00) |
| cFinADG | conv | 0.00 (0.00) | 0.18 (0.05) |
| cFinHS | conv | 0.01 (0.02) | 0.06 (0.05) |
| cFinTRT | conv | 0.00 (0.00) | 0.06 (0.05) |
| AllTRT | Not conv |  |  |
| cFinMOR | conv | 0.00 (0.00) | 0.00 (0.00) |
| AllMOR | conv | 0.00 (0.00) | 0.00 (0.00) |
| ADFI | conv | 0.00 (0.00) | 0.16 (0.05) |
| FCR | conv | 0.02 (0.03) | 0.26 (0.05) |
| RFI | conv | 0.00 (0.00) | 0.13 (0.05) |
| CWT | conv | 0.00 (0.01) | 0.28 (0.05) |
| DRS | conv | 0.00 (0.00) | 0.21 (0.05) |
| LYD | conv | 0.04 (0.03) | 0.25 (0.06) |
| CLD | conv | 0.01 (0.02) | 0.13 (0.06) |
| CBF | conv | 0.02 (0.03) | 0.25 (0.06) |

Supplemental Table 4. Estimates of the proportion of phenotypic variance explained by omics relationship matrices ($\hat{h}^{2}$) and litter effects ($c^{2}$) for the metabolome BLUP model.

| 1. **Trait** | **converge** | **h^2^_M_ (SE)** | **c^2^ (SE)** |
| --- | --- | --- | --- |
| qNurADG | conv | 0.13 (0.04) | 0.19 (0.04) |
| qNurHS1 | conv | 0.04 (0.02) | 0.00 (0.00) |
| qNurHS2 | conv | 0.06 (0.03) | 0.05 (0.05) |
| cNurADG | conv | 0.02 (0.01) | 0.11 (0.04) |
| cNurHS | conv | 0.02 (0.02) | 0.03 (0.04) |
| cNurTRT | conv | 0.00 (0.00) | 0.04 (0.04) |
| cNurMOR | conv | 0.00 (0.00) | 0.00 (0.00) |
| cFinADG | conv | 0.02 (0.02) | 0.18 (0.05) |
| cFinHS | conv | 0.00 (0.01) | 0.06 (0.05) |
| cFinTRT | conv | 0.00 (0.00) | 0.05 (0.05) |
| AllTRT | conv | 0.00 (0.00) | 0.03 (0.05) |
| cFinMOR | conv | 0.01 (0.02) | 0.00 (0.00) |
| AllMOR | conv | 0.01 (0.01) | 0.00 (0.00) |
| ADFI | conv | 0.03 (0.02) | 0.16 (0.05) |
| FCR | conv | 0.02 (0.02) | 0.26 (0.05) |
| RFI | conv | 0.01 (0.01) | 0.13 (0.05) |
| CWT | conv | 0.01 (0.02) | 0.28 (0.05) |
| DRS | conv | 0.00 (0.00) | 0.21 (0.05) |
| LYD | conv | 0.02 (0.02) | 0.26 (0.06) |
| CLD | conv | 0.01 (0.02) | 0.12 (0.06) |
| CBF | conv | 0.02 (0.02) | 0.26 (0.06) |

Supplemental Table 5. Estimates of the proportion of phenotypic variance explained by omics relationship matrices ($\hat{h}^{2}$) and litter effects ($c^{2}$) for the GTBLUP model.

| **Trait** | **converge** | **h^2^_G_ (SE)** | **h^2^_T_ (SE)** | **h^2^_GT_ (SE)** | **c^2^ (SE)** |
| --- | --- | --- | --- | --- | --- |
| qNurADG | conv | 0.21 (0.09) | 0.28 (0.09) | 0.49 (0.12) | 0.10 (0.05) |
| qNurHS1 | conv | 0.07 (0.08) | 0.09 (0.06) | 0.16 (0.09) | 0.00 (0.00) |
| qNurHS2 | conv | 0.03 (0.08) | 0.04 (0.03) | 0.07 (0.08) | 0.04 (0.05) |
| cNurADG | conv | 0.30 (0.08) | 0.02 (0.02) | 0.32 (0.09) | 0.04 (0.04) |
| cNurHS | conv | 0.06 (0.08) | 0.00 (0.00) | 0.06 (0.08) | 0.02 (0.04) |
| cNurTRT | conv | 0.18 (0.08) | 0.00 (0.00) | 0.18 (0.08) | 0.00 (0.04) |
| cNurMOR | conv | 0.14 (0.08) | 0.02 (0.02) | 0.16 (0.08) | 0.00 (0.00) |
| cFinADG | conv | 0.20 (0.09) | 0.00 (0.00) | 0.20 (0.09) | 0.12 (0.06) |
| cFinHS | conv | 0.01 (0.07) | 0.01 (0.02) | 0.02 (0.08) | 0.06 (0.05) |
| cFinTRT | conv | 0.00 (0.00) | 0.01 (0.02) | 0.01 (0.02) | 0.05 (0.05) |
| AllTRT | conv | 0.15 (0.09) | 0.00 (0.02) | 0.16 (0.09) | 0.00 (0.00) |
| cFinMOR | conv | 0.00 (0.00) | 0.01 (0.02) | 0.01 (0.02) | 0.00 (0.00) |
| AllMOR | conv | 0.09 (0.07) | 0.02 (0.02) | 0.11 (0.07) | 0.00 (0.00) |
| ADFI | conv | 0.37 (0.11) | 0.00 (0.02) | 0.38 (0.11) | 0.05 (0.05) |
| FCR | conv | 0.35 (0.11) | 0.10 (0.06) | 0.45 (0.11) | 0.18 (0.06) |
| RFI | conv | 0.68 (0.09) | 0.00 (0.01) | 0.68 (0.09) | 0.00 (0.00) |
| CWT | Not conv |  |  |  |  |
| DRS | conv | 0.30 (0.13) | 0.00 (0.00) | 0.30 (0.13) | 0.10 (0.07) |
| LYD | conv | 0.71 (0.11) | 0.02 (0.03) | 0.73 (0.11) | 0.02 (0.05) |
| CLD | conv | 0.43 (0.11) | 0.01 (0.03) | 0.44 (0.11) | 0.00 (0.00) |
| CBF | conv | 0.72 (0.11) | 0.02 (0.02) | 0.74 (0.11) | 0.03 (0.05) |

Supplemental Table 6. Estimates of the proportion of phenotypic variance explained by omics relationship matrices ($\hat{h}^{2}$) and litter effects ($c^{2}$) for the GTBLUP mean model.

| **Trait** | **converge** | **h^2^_GT_mean_ (SE)** | **c^2^ (SE)** |
| --- | --- | --- | --- |
| qNurADG | conv | 0.49 (0.11) | 0.10 (0.05) |
| qNurHS1 | conv | 0.17 (0.09) | 0.00 (0.00) |
| qNurHS2 | conv | 0.07 (0.06) | 0.04 (0.05) |
| cNurADG | conv | 0.30 (0.09) | 0.07 (0.04) |
| cNurHS | conv | 0.01 (0.04) | 0.03 (0.04) |
| cNurTRT | conv | 0.10 (0.07) | 0.02 (0.04) |
| cNurMOR | conv | 0.09 (0.07) | 0.00 (0.00) |
| cFinADG | conv | 0.08 (0.08) | 0.16 (0.06) |
| cFinHS | conv | 0.01 (0.05) | 0.06 (0.05) |
| cFinTRT | conv | 0.01 (0.04) | 0.05 (0.05) |
| AllTRT | conv | 0.07 (0.08) | 0.02 (0.05) |
| cFinMOR | conv | 0.01 (0.03) | 0.00 (0.00) |
| AllMOR | conv | 0.06 (0.05) | 0.00 (0.00) |
| ADFI | conv | 0.27 (0.12) | 0.10 (0.05) |
| FCR | conv | 0.45 (0.12) | 0.20 (0.05) |
| RFI | conv | 0.70 (0.12) | 0.01 (0.04) |
| CWT | conv | 0.73 (0.14) | 0.10 (0.05) |
| DRS | conv | 0.00 (0.00) | 0.21 (0.05) |
| LYD | conv | 0.75 (0.13) | 0.08 (0.05) |
| CLD | conv | 0.43 (0.15) | 0.04 (0.06) |
| CBF | conv | 0.70 (0.13) | 0.09 (0.05) |

Supplemental Table 7. Estimates of the proportion of phenotypic variance explained by omics relationship matrices ($\hat{h}^{2}$) and litter effects ($c^{2}$) for the GPBLUP model.

| **Trait** | **converge** | **h^2^_G_ (SE)** | **h^2^_P_ (SE)** | **h^2^_GP_ (SE)** | **c^2^ (SE)** |
| --- | --- | --- | --- | --- | --- |
| **qNurADG** | conv | 0.2 (0.08) | 0.21 (0.06) | 0.41 (0.09) | 0.1 (0.05) |
| **qNurHS1** | conv | 0.09 (0.08) | 0.09 (0.05) | 0.18 (0.09) | 0 (0) |
| **qNurHS2** | conv | 0.01 (0.07) | 0.02 (0.02) | 0.04 (0.08) | 0.04 (0.05) |
| **cNurADG** | Not conv |  |  |  |  |
| **cNurHS** | conv | 0.06 (0.07) | 0.02 (0.02) | 0.08 (0.08) | 0.02 (0.04) |
| **cNurTRT** | conv | 0.18 (0.08) | 0 (0.01) | 0.18 (0.08) | 0 (0.04) |
| **cNurMOR** | Not conv |  |  |  |  |
| **cFinADG** | conv | 0.2 (0.09) | 0 (0) | 0.2 (0.09) | 0.12 (0.06) |
| **cFinHS** | Not conv |  |  |  |  |
| **cFinTRT** | conv | 0 (0) | 0 (0) | 0 (0) | 0.05 (0.05) |
| **AllTRT** | conv | 0.15 (0.09) | 0 (0) | 0.15 (0.09) | 0 (0) |
| **cFinMOR** | conv |  |  |  |  |
| **AllMOR** | conv | 0.11 (0.07) | 0 (0) | 0.11 (0.07) | 0 (0) |
| **ADFI** | conv | 0.37 (0.11) | 0 (0) | 0.37 (0.11) | 0.05 (0.05) |
| **FCR** | conv | 0.38 (0.11) | 0.03 (0.03) | 0.41 (0.11) | 0.18 (0.06) |
| **RFI** | conv | 0.68 (0.09) | 0 (0.01) | 0.68 (0.09) | 0 (0) |
| **CWT** | Not conv |  |  |  |  |
| **DRS** | conv | 0.3 (0.13) | 0 (0) | 0.3 (0.13) | 0.1 (0.07) |
| **LYD** | conv | 0.68 (0.11) | 0.03 (0.02) | 0.71 (0.11) | 0.01 (0.05) |
| **CLD** | conv | 0.42 (0.11) | 0.01 (0.02) | 0.43 (0.11) | 0 (0) |
| **CBF** | conv | 0.7 (0.11) | 0.01 (0.02) | 0.71 (0.11) | 0.02 (0.05) |

Supplemental Table 8. Estimates of the proportion of phenotypic variance explained by omics relationship matrices ($\hat{h}^{2}$) and litter effects ($c^{2}$) for the GPBLUP (mean) model.

| **Trait** | **converge** | **h^2^_GP_mean_ (SE)** | **c^2^ (SE)** |
| --- | --- | --- | --- |
| **qNurADG** | conv | 0.41 (0.08) | 0.1 (0.04) |
| **qNurHS1** | conv | 0.18 (0.08) | 0 (0) |
| **qNurHS2** | conv | 0.05 (0.04) | 0.04 (0.05) |
| **cNurADG** | conv | 0.24 (0.07) | 0.07 (0.04) |
| **cNurHS** | conv | 0.06 (0.05) | 0.03 (0.04) |
| **cNurTRT** | conv | 0.08 (0.06) | 0.03 (0.04) |
| **cNurMOR** | conv | 0 (0.01) | 0 (0) |
| **cFinADG** | conv | 0.07 (0.07) | 0.16 (0.06) |
| **cFinHS** | conv | 0.03 (0.03) | 0.06 (0.05) |
| **cFinTRT** | conv | 0 (0) | 0.05 (0.05) |
| **AllTRT** | conv | 0 (0) | 0.03 (0.05) |
| **cFinMOR** | conv | 0 (0) | 0 (0) |
| **AllMOR** | conv | 0 (0) | 0 (0) |
| **ADFI** | conv | 0.19 (0.09) | 0.11 (0.05) |
| **FCR** | conv | 0.23 (0.09) | 0.21 (0.06) |
| **RFI** | Not conv |  |  |
| **CWT** | conv | 0.02 (0.04) | 0.27 (0.05) |
| **DRS** | conv | 0 (0) | 0.21 (0.05) |
| **LYD** | conv | 0.5 (0.1) | 0.09 (0.05) |
| **CLD** | conv | 0.19 (0.1) | 0.07 (0.06) |
| **CBF** | conv | 0.46 (0.11) | 0.1 (0.05) |

Supplemental Table 9. Estimates of the proportion of phenotypic variance explained by omics relationship matrices ($\hat{h}^{2}$) and litter effects ($c^{2}$) for the GMBLUP model.

| **Trait** | **converge** | **h^2^_G_ (SE)** | **h^2^_M_ (SE)** | **h^2^_GM_ (SE)** | **c^2^ (SE)** |
| --- | --- | --- | --- | --- | --- |
| **qNurADG** | conv | 0.11 (0.08) | 0.12 (0.04) | 0.24 (0.08) | 0.15 (0.05) |
| **qNurHS1** | conv | 0.06 (0.08) | 0.04 (0.02) | 0.1 (0.08) | 0 (0) |
| **qNurHS2** | conv | 0.07 (0.08) | 0.06 (0.03) | 0.13 (0.09) | 0.06 (0.05) |
| **cNurADG** | conv | 0.3 (0.08) | 0.02 (0.01) | 0.31 (0.08) | 0.04 (0.04) |
| **cNurHS** | conv | 0.06 (0.07) | 0.02 (0.02) | 0.08 (0.07) | 0.02 (0.04) |
| **cNurTRT** | conv | 0.18 (0.08) | 0 (0) | 0.18 (0.08) | 0 (0.04) |
| **cNurMOR** | conv | 0.15 (0.08) | 0 (0) | 0.15 (0.08) | 0 (0) |
| **cFinADG** | Not conv |  |  |  |  |
| **cFinHS** | conv | 0.01 (0.07) | 0 (0.01) | 0.01 (0.08) | 0.06 (0.05) |
| **cFinTRT** | Not conv |  |  |  |  |
| **AllTRT** | conv | 0.15 (0.09) | 0 (0) | 0.15 (0.09) | 0 (0) |
| **cFinMOR** | conv | 0 (0) | 0.01 (0.02) | 0.01 (0.02) | 0 (0) |
| **AllMOR** | conv | 0.11 (0.07) | 0.01 (0.01) | 0.11 (0.07) | 0 (0) |
| **ADFI** | Not conv |  |  |  |  |
| **FCR** | Not conv |  |  |  |  |
| **RFI** | Not conv |  |  |  |  |
| **CWT** | Not conv |  |  |  |  |
| **DRS** | conv | 0.3 (0.13) | 0 (0) | 0.3 (0.13) | 0.1 (0.07) |
| **LYD** | Not conv |  |  |  |  |
| **CLD** | conv | 0.42 (0.11) | 0.01 (0.02) | 0.43 (0.11) | 0 (0) |
| **CBF** | conv | 0.71 (0.11) | 0.02 (0.02) | 0.73 (0.11) | 0.03 (0.05) |

Supplemental Table 10. Estimates of the proportion of phenotypic variance explained by omics relationship matrices ($\hat{h}^{2}$) and litter effects ($c^{2}$) for the GMBLUP (mean) model.

| **Trait** | **converge** | **h^2^_GM_mean_ (SE)** | **c^2^ (SE)** |
| --- | --- | --- | --- |
| **qNurADG** | conv | 0.25 (0.06) | 0.14 (0.04) |
| **qNurHS1** | conv | 0.09 (0.04) | 0 (0) |
| **qNurHS2** | conv | 0.12 (0.05) | 0.06 (0.05) |
| **cNurADG** | conv | 0.1 (0.05) | 0.09 (0.04) |
| **cNurHS** | conv | 0.05 (0.04) | 0.03 (0.04) |
| **cNurTRT** | conv | 0.01 (0.02) | 0.04 (0.04) |
| **cNurMOR** | conv | 0 (0.01) | 0 (0) |
| **cFinADG** | conv | 0.06 (0.04) | 0.17 (0.05) |
| **cFinHS** | conv | 0 (0.02) | 0.06 (0.05) |
| **cFinTRT** | conv | 0 (0) | 0.05 (0.05) |
| **AllTRT** | conv | 0 (0) | 0.03 (0.05) |
| **cFinMOR** | conv | 0.02 (0.03) | 0 (0) |
| **AllMOR** | conv | 0.02 (0.03) | 0 (0) |
| **ADFI** | conv | 0.12 (0.06) | 0.13 (0.05) |
| **FCR** | conv | 0.06 (0.04) | 0.24 (0.06) |
| **RFI** | Not conv |  |  |
| **CWT** | conv | 0.03 (0.04) | 0.27 (0.05) |
| **DRS** | conv | 0.02 (0.04) | 0.21 (0.06) |
| **LYD** | Not conv |  |  |
| **CLD** | conv | 0.1 (0.07) | 0.08 (0.06) |
| **CBF** | Not conv |  |  |

Supplemental Table 11. Estimates of the proportion of phenotypic variance explained by omics relationship matrices ($\hat{h}^{2}$) and litter effects ($c^{2}$) for the TPBLUP model.

| **Trait** | **converge** | **h^2^_T_ (SE)** | **h^2^_P_ (SE)** | **h^2^_TP_ (SE)** | **c^2^ (SE)** |
| --- | --- | --- | --- | --- | --- |
| **qNurADG** | conv | 0.15 (0.07) | 0.18 (0.05) | 0.33 (0.08) | 0.15 (0.04) |
| **qNurHS1** | conv | 0.08 (0.06) | 0.06 (0.04) | 0.15 (0.07) | 0.02 (0.04) |
| **qNurHS2** | conv | 0.03 (0.03) | 0.02 (0.02) | 0.05 (0.03) | 0.04 (0.05) |
| **cNurADG** | conv | 0.04 (0.04) | 0 (0.01) | 0.04 (0.04) | 0.11 (0.04) |
| **cNurHS** | conv | 0 (0) | 0.03 (0.02) | 0.03 (0.02) | 0.03 (0.04) |
| **cNurTRT** | conv | 0 (0) | 0 (0.01) | 0 (0.01) | 0.04 (0.04) |
| **cNurMOR** | conv | 0.02 (0.03) | 0 (0) | 0.02 (0.03) | 0 (0) |
| **cFinADG** | Not conv |  |  |  |  |
| **cFinHS** | conv | 0 (0.02) | 0.01 (0.02) | 0.01 (0.03) | 0.06 (0.05) |
| **cFinTRT** | Not conv |  |  |  |  |
| **AllTRT** | conv | 0.01 (0.02) | 0 (0) | 0.01 (0.02) | 0.03 (0.05) |
| **cFinMOR** | conv | 0.01 (0.02) | 0 (0) | 0.01 (0.02) | 0 (0) |
| **AllMOR** | conv | 0.02 (0.02) | 0 (0) | 0.02 (0.02) | 0 (0) |
| **ADFI** | conv | 0.01 (0.02) | 0 (0) | 0.01 (0.02) | 0.16 (0.05) |
| **FCR** | Not conv |  |  |  |  |
| **RFI** | Not conv |  |  |  |  |
| **CWT** | Not conv |  |  |  |  |
| **DRS** | conv | 0 (0) | 0 (0) | 0 (0) | 0.21 (0.05) |
| **LYD** | conv | 0.03 (0.03) | 0.04 (0.03) | 0.07 (0.05) | 0.25 (0.06) |
| **CLD** | conv | 0.02 (0.04) | 0.01 (0.02) | 0.03 (0.05) | 0.13 (0.06) |
| **CBF** | conv | 0.03 (0.03) | 0.02 (0.03) | 0.05 (0.04) | 0.25 (0.06) |

Supplemental Table 12. Estimates of the proportion of phenotypic variance explained by omics relationship matrices ($\hat{h}^{2}$) and litter effects ($c^{2}$) for the TPBLUP (mean) model.

| **Trait** | **converge** | **h^2^_TP_mean_ (SE)** | **c^2^ (SE)** |
| --- | --- | --- | --- |
| **qNurADG** | conv | 0.34 (0.08) | 0.15 (0.04) |
| **qNurHS1** | conv | 0.15 (0.06) | 0.02 (0.04) |
| **qNurHS2** | conv | 0.04 (0.03) | 0.04 (0.05) |
| **cNurADG** | conv | 0.05 (0.04) | 0.11 (0.04) |
| **cNurHS** | conv | 0.03 (0.03) | 0.03 (0.04) |
| **cNurTRT** | conv | 0 (0.02) | 0.04 (0.04) |
| **cNurMOR** | conv | 0.01 (0.02) | 0 (0) |
| **cFinADG** | conv | 0 (0) | 0.18 (0.05) |
| **cFinHS** | conv | 0.02 (0.03) | 0.06 (0.05) |
| **cFinTRT** | conv | 0 (0) | 0.05 (0.05) |
| **AllTRT** | conv | 0 (0) | 0.03 (0.05) |
| **cFinMOR** | conv | 0 (0) | 0 (0) |
| **AllMOR** | conv | 0.02 (0.02) | 0 (0) |
| **ADFI** | conv | 0 (0.02) | 0.16 (0.05) |
| **FCR** | conv | 0.11 (0.06) | 0.26 (0.05) |
| **RFI** | conv | 0 (0) | 0.13 (0.05) |
| **CWT** | conv | 0 (0) | 0.28 (0.05) |
| **DRS** | conv | 0 (0) | 0.21 (0.05) |
| **LYD** | conv | 0.07 (0.05) | 0.26 (0.06) |
| **CLD** | conv | 0.02 (0.04) | 0.13 (0.06) |
| **CBF** | conv | 0.05 (0.04) | 0.25 (0.06) |

Supplemental Table 13. Estimates of the proportion of phenotypic variance explained by omics relationship matrices ($\hat{h}^{2}$) and litter effects ($c^{2}$) for the TMBLUP model.

| **Trait** | **converge** | **h^2^_T_ (SE)** | **h^2^_M_ (SE)** | **h^2^_TM_ (SE)** | **c^2^ (SE)** |
| --- | --- | --- | --- | --- | --- |
| **qNurADG** | conv | 0.04 (0.04) | 0.13 (0.04) | 0.17 (0.05) | 0.18 (0.04) |
| **qNurHS1** | conv | 0.04 (0.04) | 0.04 (0.02) | 0.08 (0.04) | 0 (0) |
| **qNurHS2** | conv | 0 (0) | 0.06 (0.03) | 0.06 (0.03) | 0.07 (0.05) |
| **cNurADG** | conv | 0.02 (0.03) | 0.02 (0.01) | 0.03 (0.03) | 0.11 (0.04) |
| **cNurHS** | conv | 0 (0) | 0.02 (0.02) | 0.02 (0.02) | 0.03 (0.04) |
| **cNurTRT** | conv | 0 (0) | 0 (0) | 0 (0) | 0.04 (0.04) |
| **cNurMOR** | conv | 0.02 (0.03) | 0 (0) | 0.02 (0.03) | 0 (0) |
| **cFinADG** | conv | 0 (0) | 0.02 (0.02) | 0.02 (0.02) | 0.18 (0.05) |
| **cFinHS** | conv | 0.01 (0.02) | 0 (0.01) | 0.01 (0.03) | 0.06 (0.05) |
| **cFinTRT** | conv | 0.01 (0.02) | 0 (0) | 0.01 (0.02) | 0.05 (0.05) |
| **AllTRT** | conv | 0.01 (0.02) | 0 (0) | 0.01 (0.02) | 0.03 (0.05) |
| **cFinMOR** | conv | 0.01 (0.02) | 0.01 (0.02) | 0.02 (0.02) | 0 (0) |
| **AllMOR** | conv | 0.02 (0.02) | 0 (0.01) | 0.03 (0.02) | 0 (0) |
| **ADFI** | conv | 0.01 (0.02) | 0.03 (0.02) | 0.04 (0.03) | 0.16 (0.05) |
| **FCR** | conv | 0.11 (0.07) | 0.02 (0.02) | 0.13 (0.07) | 0.26 (0.05) |
| **RFI** | conv | 0 (0.02) | 0.01 (0.01) | 0.01 (0.02) | 0.13 (0.05) |
| **CWT** | Not conv |  |  |  |  |
| **DRS** | conv | 0 (0) | 0 (0) | 0 (0) | 0.21 (0.05) |
| **LYD** | conv | 0.03 (0.03) | 0.02 (0.02) | 0.05 (0.04) | 0.26 (0.06) |
| **CLD** | conv | 0.03 (0.05) | 0.01 (0.02) | 0.04 (0.05) | 0.13 (0.06) |
| **CBF** | conv | 0.02 (0.03) | 0.02 (0.02) | 0.04 (0.04) | 0.26 (0.06) |

Supplemental Table 14. Estimates of the proportion of phenotypic variance explained by omics relationship matrices ($\hat{h}^{2}$) and litter effects ($c^{2}$) for the TMBLUP (mean) model.

| **Trait** | **converge** | **h^2^_TM_mean_ (SE)** | **c^2^ (SE)** |
| --- | --- | --- | --- |
| **qNurADG** | conv | 0.21 (0.05) | 0.17 (0.04) |
| **qNurHS1** | conv | 0.08 (0.03) | 0 (0) |
| **qNurHS2** | conv | 0.07 (0.03) | 0.07 (0.05) |
| **cNurADG** | conv | 0.03 (0.02) | 0.11 (0.04) |
| **cNurHS** | conv | 0.03 (0.02) | 0.03 (0.04) |
| **cNurTRT** | conv | 0 (0) | 0.04 (0.04) |
| **cNurMOR** | conv | 0.01 (0.01) | 0 (0) |
| **cFinADG** | conv | 0.02 (0.02) | 0.18 (0.05) |
| **cFinHS** | conv | 0 (0.02) | 0.06 (0.05) |
| **cFinTRT** | conv | 0 (0) | 0.05 (0.05) |
| **AllTRT** | conv | 0 (0) | 0.03 (0.05) |
| **cFinMOR** | conv | 0.02 (0.02) | 0 (0) |
| **AllMOR** | conv | 0.02 (0.02) | 0 (0) |
| **ADFI** | conv | 0.05 (0.03) | 0.16 (0.05) |
| **FCR** | conv | 0.06 (0.04) | 0.25 (0.05) |
| **RFI** | conv | 0.01 (0.02) | 0.13 (0.05) |
| **CWT** | conv | 0.03 (0.03) | 0.27 (0.05) |
| **DRS** | conv | 0 (0) | 0.21 (0.05) |
| **LYD** | conv | 0.04 (0.03) | 0.26 (0.06) |
| **CLD** | conv | 0.03 (0.03) | 0.12 (0.06) |
| **CBF** | conv | 0.04 (0.03) | 0.26 (0.06) |

Supplemental Table 15. Estimates of the proportion of phenotypic variance explained by omics relationship matrices ($\hat{h}^{2}$) and litter effects ($c^{2}$) for the PMBLUP model.

| **Trait** | **converge** | **h^2^_P_ (SE)** | **h^2^_M_ (SE)** | **h^2^_PM_ (SE)** | **c^2^ (SE)** |
| --- | --- | --- | --- | --- | --- |
| **qNurADG** | conv | 0.1 (0.05) | 0.1 (0.03) | 0.21 (0.05) | 0.18 (0.04) |
| **qNurHS1** | conv | 0 (0.01) | 0.04 (0.02) | 0.04 (0.02) | 0 (0) |
| **qNurHS2** | conv | 0.01 (0.01) | 0.06 (0.03) | 0.06 (0.03) | 0.07 (0.05) |
| **cNurADG** | Not conv |  |  |  |  |
| **cNurHS** | conv | 0.01 (0.02) | 0.02 (0.02) | 0.03 (0.02) | 0.03 (0.04) |
| **cNurTRT** | conv | 0 (0.01) | 0 (0) | 0 (0.01) | 0.04 (0.04) |
| **cNurMOR** | conv | 0 (0.01) | 0 (0) | 0 (0.01) | 0 (0) |
| **cFinADG** | conv | 0 (0) | 0.02 (0.02) | 0.02 (0.02) | 0.18 (0.05) |
| **cFinHS** | conv | 0.01 (0.02) | 0 (0.01) | 0.01 (0.02) | 0.06 (0.05) |
| **cFinTRT** | conv | 0 (0) | 0 (0) | 0 (0) | 0.05 (0.05) |
| **AllTRT** | conv | 0 (0) | 0 (0) | 0 (0) | 0.03 (0.05) |
| **cFinMOR** | conv | 0 (0) | 0.01 (0.02) | 0.01 (0.02) | 0 (0) |
| **AllMOR** | conv | 0 (0) | 0.01 (0.01) | 0.01 (0.01) | 0 (0) |
| **ADFI** | conv | 0 (0) | 0.03 (0.02) | 0.03 (0.02) | 0.16 (0.05) |
| **FCR** | conv | 0.02 (0.03) | 0.02 (0.02) | 0.04 (0.03) | 0.26 (0.05) |
| **RFI** | Not conv |  |  |  |  |
| **CWT** | conv | 0 (0) | 0.01 (0.02) | 0.01 (0.02) | 0.28 (0.05) |
| **DRS** | Not conv |  |  |  |  |
| **LYD** | conv | 0.03 (0.03) | 0.01 (0.02) | 0.05 (0.03) | 0.25 (0.06) |
| **CLD** | conv | 0.01 (0.02) | 0.01 (0.02) | 0.02 (0.03) | 0.12 (0.06) |
| **CBF** | conv | 0.02 (0.02) | 0.02 (0.02) | 0.04 (0.03) | 0.25 (0.06) |

Supplemental Table 16. Estimates of the proportion of phenotypic variance explained by omics relationship matrices ($\hat{h}^{2}$) and litter effects ($c^{2}$) for the PMBLUP (mean) model.

| **Trait** | **converge** | **h^2^_PM_mean_ (SE)** | **c^2^ (SE)** |
| --- | --- | --- | --- |
| **qNurADG** | conv | 0.21 (0.05) | 0.18 (0.04) |
| **qNurHS1** | conv | 0.07 (0.03) | 0 (0.04) |
| **qNurHS2** | conv | 0.07 (0.03) | 0.06 (0.05) |
| **cNurADG** | conv | 0.03 (0.02) | 0.11 (0.04) |
| **cNurHS** | conv | 0.04 (0.02) | 0.03 (0.04) |
| **cNurTRT** | conv | 0 (0.01) | 0.04 (0.04) |
| **cNurMOR** | conv | 0 (0.01) | 0 (0) |
| **cFinADG** | Not conv |  |  |
| **cFinHS** | conv | 0.01 (0.02) | 0.06 (0.05) |
| **cFinTRT** | Not conv |  |  |
| **AllTRT** | conv | 0 (0) | 0.03 (0.05) |
| **cFinMOR** | conv | 0 (0.02) | 0 (0) |
| **AllMOR** | Not conv |  |  |
| **ADFI** | Not conv |  |  |
| **FCR** | conv | 0.04 (0.03) | 0.26 (0.05) |
| **RFI** | conv | 0.01 (0.02) | 0.13 (0.05) |
| **CWT** | conv | 0.01 (0.02) | 0.28 (0.05) |
| **DRS** | conv | 0 (0.01) | 0.21 (0.05) |
| **LYD** | conv | 0.04 (0.03) | 0.25 (0.06) |
| **CLD** | conv | 0.02 (0.03) | 0.12 (0.06) |
| **CBF** | conv | 0.03 (0.03) | 0.25 (0.06) |

Supplemental Table 17. Estimates of the proportion of phenotypic variance explained by omics relationship matrices ($\hat{h}^{2}$) and litter effects ($c^{2}$) for the GTPBLUP model.

| **Trait** | **conv6erge** | **h^2^_G_ (SE)** | **h^2^_T_ (SE)** | **h^2^_P_ (SE)** | **h^2^_GTP_ (SE)** | **c^2^ (SE)** |
| --- | --- | --- | --- | --- | --- | --- |
| **qNurADG** | Not conv |  |  |  |  |  |
| **qNurHS1** | conv | 0.07 (0.09) | 0.08 (0.05) | 0.06 (0.04) | 0.21 (0.1) | 0.01 (0.05) |
| **qNurHS2** | Not conv |  |  |  |  |  |
| **cNurADG** | conv | 0.3 (0.08) | 0.02 (0.02) | 0 (0) | 0.32 (0.09) | 0.04 (0.04) |
| **cNurHS** | Not conv |  |  |  |  |  |
| **cNurTRT** | conv | 0.18 (0.08) | 0 (0) | 0 (0.01) | 0.18 (0.08) | 0 (0.04) |
| **cNurMOR** | conv | 0.14 (0.08) | 0.02 (0.02) | 0 (0) | 0.16 (0.08) | 0 (0) |
| **cFinADG** | conv | 0.2 (0.09) | 0 (0) | 0 (0) | 0.2 (0.09) | 0.12 (0.06) |
| **cFinHS** | Not conv |  |  |  |  |  |
| **cFinTRT** | conv | 0 (0) | 0.01 (0.02) | 0 (0) | 0.01 (0.02) | 0.05 (0.05) |
| **AllTRT** | conv | 0.15 (0.09) | 0 (0.02) | 0 (0) | 0.16 (0.09) | 0 (0) |
| **cFinMOR** | conv | 0 (0) | 0.01 (0.02) | 0 (0) | 0.01 (0.02) | 0 (0) |
| **AllMOR** | Not conv |  |  |  |  |  |
| **ADFI** | Not conv |  |  |  |  |  |
| **FCR** | conv | 0.36 (0.11) | 0.09 (0.06) | 0.02 (0.03) | 0.47 (0.11) | 0.18 (0.06) |
| **RFI** | conv | 0.68 (0.09) | 0 (0.01) | 0 (0.01) | 0.68 (0.09) | 0 (0) |
| **CWT** | Not conv |  |  |  |  |  |
| **DRS** | conv | 0.3 (0.13) | 0 (0) | 0 (0) | 0.3 (0.13) | 0.1 (0.07) |
| **LYD** | conv | 0.68 (0.11) | 0.03 (0.03) | 0.03 (0.03) | 0.73 (0.11) | 0.01 (0.05) |
| **CLD** | conv | 0.43 (0.11) | 0.01 (0.03) | 0.01 (0.01) | 0.44 (0.11) | 0 (0) |
| **CBF** | conv | 0.7 (0.11) | 0.02 (0.03) | 0.02 (0.02) | 0.73 (0.11) | 0.02 (0.05) |

Supplemental Table 18. Estimates of the proportion of phenotypic variance explained by omics relationship matrices ($\hat{h}^{2}$) and litter effects ($c^{2}$) for the GTPBLUP (mean) model.

| **Trait** | **converge** | **h^2^_GTP_mean_ (SE)** | **c^2^ (SE)** |
| --- | --- | --- | --- |
| **qNurADG** | conv | 0.5 (0.09) | 0.09 (0.04) |
| **qNurHS1** | conv | 0.21 (0.09) | 0.01 (0.04) |
| **qNurHS2** | conv | 0.06 (0.04) | 0.04 (0.05) |
| **cNurADG** | conv | 0.22 (0.08) | 0.09 (0.04) |
| **cNurHS** | conv | 0.05 (0.04) | 0.03 (0.04) |
| **cNurTRT** | conv | 0.05 (0.05) | 0.03 (0.04) |
| **cNurMOR** | conv | 0.02 (0.03) | 0 (0) |
| **cFinADG** | conv | 0 (0) | 0.18 (0.05) |
| **cFinHS** | conv | 0.03 (0.04) | 0.06 (0.05) |
| **cFinTRT** | conv | 0 (0) | 0.05 (0.05) |
| **AllTRT** | conv | 0 (0) | 0.03 (0.05) |
| **cFinMOR** | conv | 0 (0) | 0 (0) |
| **AllMOR** | conv | 0.04 (0.04) | 0 (0) |
| **ADFI** | conv | 0.1 (0.08) | 0.14 (0.05) |
| **FCR** | conv | 0.34 (0.11) | 0.21 (0.05) |
| **RFI** | Not conv |  |  |
| **CWT** | Not conv |  |  |
| **DRS** | conv | 0 (0) | 0.21 (0.05) |
| **LYD** | conv | 0.51 (0.12) | 0.12 (0.05) |
| **CLD** | conv | 0.18 (0.1) | 0.09 (0.06) |
| **CBF** | conv | 0.43 (0.12) | 0.13 (0.06) |

Supplemental Table 19. Estimates of the proportion of phenotypic variance explained by omics relationship matrices ($\hat{h}^{2}$) and litter effects ($c^{2}$) for the GTMBLUP model.

| **Trait** | **converge** | **h^2^_G_ (SE)** | **h^2^_T_ (SE)** | **h^2^_M_ (SE)** | **h^2^_GTM_ (SE)** | **c^2^ (SE)** |
| --- | --- | --- | --- | --- | --- | --- |
| **qNurADG** | conv | 0.1 (0.08) | 0.03 (0.03) | 0.12 (0.04) | 0.26 (0.09) | 0.14 (0.05) |
| **qNurHS1** | conv | 0.06 (0.08) | 0.04 (0.04) | 0.04 (0.02) | 0.13 (0.08) | 0 (0) |
| **qNurHS2** | conv | 0.07 (0.08) | 0 (0) | 0.06 (0.03) | 0.13 (0.09) | 0.06 (0.05) |
| **cNurADG** | conv | 0.3 (0.08) | 0.01 (0.02) | 0.01 (0.01) | 0.32 (0.08) | 0.04 (0.04) |
| **cNurHS** | Not conv |  |  |  |  |  |
| **cNurTRT** | conv | 0.18 (0.08) | 0 (0) | 0 (0) | 0.18 (0.08) | 0 (0.04) |
| **cNurMOR** | conv | 0.14 (0.08) | 0.02 (0.02) | 0 (0) | 0.16 (0.08) | 0 (0) |
| **cFinADG** | conv | 0.2 (0.09) | 0 (0) | 0.01 (0.01) | 0.21 (0.09) | 0.12 (0.06) |
| **cFinHS** | conv | 0.01 (0.07) | 0.01 (0.02) | 0 (0.01) | 0.02 (0.08) | 0.06 (0.05) |
| **cFinTRT** | conv | 0 (0) | 0.01 (0.02) | 0 (0) | 0.01 (0.02) | 0.05 (0.05) |
| **AllTRT** | Not conv |  |  |  |  |  |
| **cFinMOR** | conv | 0 (0) | 0.01 (0.02) | 0.01 (0.02) | 0.02 (0.02) | 0 (0) |
| **AllMOR** | conv | 0.09 (0.07) | 0.02 (0.02) | 0 (0.01) | 0.12 (0.07) | 0 (0) |
| **ADFI** | conv | 0.36 (0.11) | 0 (0.02) | 0.02 (0.02) | 0.39 (0.11) | 0.05 (0.05) |
| **FCR** | conv | 0.34 (0.11) | 0.09 (0.06) | 0.01 (0.01) | 0.44 (0.11) | 0.18 (0.06) |
| **RFI** | conv | 0.68 (0.09) | 0 (0.01) | 0 (0) | 0.68 (0.09) | 0 (0) |
| **CWT** | Not conv |  |  |  |  |  |
| **DRS** | conv | 0.3 (0.13) | 0 (0) | 0 (0) | 0.3 (0.13) | 0.1 (0.07) |
| **LYD** | conv | 0.71 (0.11) | 0.03 (0.03) | 0.02 (0.02) | 0.75 (0.11) | 0.01 (0.05) |
| **CLD** | Not conv |  |  |  |  |  |
| **CBF** | Not conv |  |  |  |  |  |

Supplemental Table 20. Estimates of the proportion of phenotypic variance explained by omics relationship matrices ($\hat{h}^{2}$) and litter effects ($c^{2}$) for the GTMBLUP (mean) model.

| **Trait** | **converge** | **h^2^_GTM_mean_ (SE)** | **c^2^ (SE)** |
| --- | --- | --- | --- |
| **qNurADG** | conv | 0.31 (0.07) | 0.14 (0.04) |
| **qNurHS1** | conv | 0.11 (0.05) | 0 (0) |
| **qNurHS2** | conv | 0.11 (0.05) | 0.06 (0.05) |
| **cNurADG** | conv | 0.1 (0.05) | 0.1 (0.04) |
| **cNurHS** | conv | 0.05 (0.04) | 0.02 (0.04) |
| **cNurTRT** | Not conv |  |  |
| **cNurMOR** | conv | 0.01 (0.02) | 0 (0) |
| **cFinADG** | conv | 0.05 (0.04) | 0.17 (0.05) |
| **cFinHS** | conv | 0.01 (0.03) | 0.06 (0.05) |
| **cFinTRT** | conv | 0 (0) | 0.05 (0.05) |
| **AllTRT** | conv | 0 (0) | 0.03 (0.05) |
| **cFinMOR** | conv | 0.02 (0.03) | 0 (0) |
| **AllMOR** | conv | 0.04 (0.03) | 0 (0) |
| **ADFI** | conv | 0.12 (0.06) | 0.14 (0.05) |
| **FCR** | conv | 0.13 (0.06) | 0.24 (0.05) |
| **RFI** | conv | 0.11 (0.07) | 0.1 (0.05) |
| **CWT** | conv | 0.07 (0.06) | 0.26 (0.05) |
| **DRS** | conv | 0 (0) | 0.21 (0.05) |
| **LYD** | Not conv |  |  |
| **CLD** | conv | 0.12 (0.07) | 0.1 (0.06) |
| **CBF** | conv | 0.21 (0.09) | 0.2 (0.06) |

Supplemental Table 21. Estimates of the proportion of phenotypic variance explained by omics relationship matrices ($\hat{h}^{2}$) and litter effects ($c^{2}$) for the GPMBLUP model.

| **Trait** | **converge** | **h^2^_G_ (SE)** | **h^2^_P_ (SE)** | **h^2^_M_ (SE)** | **h^2^_GPM_ (SE)** | **c^2^ (SE)** |
| --- | --- | --- | --- | --- | --- | --- |
| **qNurADG** | conv | 0.1 (0.08) | 0.1 (0.05) | 0.1 (0.03) | 0.3 (0.09) | 0.14 (0.05) |
| **qNurHS1** | Not conv |  |  |  |  |  |
| **qNurHS2** | conv | 0.06 (0.08) | 0.01 (0.01) | 0.06 (0.03) | 0.12 (0.09) | 0.06 (0.05) |
| **cNurADG** | Not conv |  |  |  |  |  |
| **cNurHS** | conv | 0.05 (0.07) | 0.01 (0.02) | 0.02 (0.02) | 0.09 (0.08) | 0.02 (0.04) |
| **cNurTRT** | conv | 0.18 (0.08) | 0 (0.01) | 0 (0) | 0.18 (0.08) | 0 (0.04) |
| **cNurMOR** | conv | 0.15 (0.08) | 0 (0) | 0 (0) | 0.15 (0.08) | 0 (0) |
| **cFinADG** | conv | 0.2 (0.09) | 0 (0) | 0.01 (0.01) | 0.21 (0.09) | 0.12 (0.06) |
| **cFinHS** | conv | 0.01 (0.07) | 0.01 (0.02) | 0 (0.01) | 0.02 (0.08) | 0.06 (0.05) |
| **cFinTRT** | conv | 0 (0) | 0 (0) | 0 (0) | 0 (0) | 0.05 (0.05) |
| **AllTRT** | conv | 0.15 (0.09) | 0 (0) | 0 (0) | 0.15 (0.09) | 0 (0) |
| **cFinMOR** | conv | 0 (0) | 0 (0) | 0.01 (0.02) | 0.01 (0.02) | 0 (0) |
| **AllMOR** | conv | 0.11 (0.07) | 0 (0) | 0.01 (0.01) | 0.11 (0.07) | 0 (0) |
| **ADFI** | conv | 0.36 (0.11) | 0 (0) | 0.02 (0.02) | 0.38 (0.11) | 0.05 (0.05) |
| **FCR** | conv | 0.37 (0.11) | 0.03 (0.03) | 0.02 (0.01) | 0.41 (0.11) | 0.17 (0.06) |
| **RFI** | conv | 0.68 (0.09) | 0 (0.01) | 0 (0) | 0.68 (0.09) | 0 (0) |
| **CWT** | conv | 0.24 (0.13) | 0 (0) | 0 (0.01) | 0.25 (0.13) | 0.17 (0.07) |
| **DRS** | Not conv |  |  |  |  |  |
| **LYD** | conv | 0.68 (0.11) | 0.02 (0.02) | 0.01 (0.01) | 0.72 (0.11) | 0.01 (0.05) |
| **CLD** | conv | 0.42 (0.11) | 0 (0.01) | 0.01 (0.02) | 0.43 (0.11) | 0 (0) |
| **CBF** | conv | 0.7 (0.11) | 0.01 (0.02) | 0.01 (0.01) | 0.72 (0.11) | 0.02 (0.05) |

Supplemental Table 22. Estimates of the proportion of phenotypic variance explained by omics relationship matrices ($\hat{h}^{2}$) and litter effects ($c^{2}$) for the GPMBLUP (mean) model.

| **Trait** | **converge** | **h^2^_GPM_mean_ (SE)** | **c^2^ (SE)** |
| --- | --- | --- | --- |
| **qNurADG** | conv | 0.3 (0.06) | 0.14 (0.04) |
| **qNurHS1** | conv | 0.12 (0.05) | 0 (0) |
| **qNurHS2** | conv | 0.1 (0.04) | 0.06 (0.05) |
| **cNurADG** | conv | 0.09 (0.05) | 0.1 (0.04) |
| **cNurHS** | conv | 0.06 (0.04) | 0.03 (0.04) |
| **cNurTRT** | conv | 0.01 (0.02) | 0.04 (0.04) |
| **cNurMOR** | conv | 0 (0.01) | 0 (0) |
| **cFinADG** | conv | 0.05 (0.04) | 0.17 (0.05) |
| **cFinHS** | conv | 0.02 (0.02) | 0.06 (0.05) |
| **cFinTRT** | conv | 0 (0) | 0.05 (0.05) |
| **AllTRT** | conv | 0 (0) | 0.03 (0.05) |
| **cFinMOR** | conv | 0 (0) | 0 (0) |
| **AllMOR** | conv | 0 (0) | 0 (0) |
| **ADFI** | conv | 0.1 (0.05) | 0.14 (0.05) |
| **FCR** | conv | 0.09 (0.05) | 0.24 (0.05) |
| **RFI** | conv | 0.08 (0.06) | 0.11 (0.05) |
| **CWT** | conv | 0.02 (0.03) | 0.27 (0.05) |
| **DRS** | conv | 0 (0) | 0.21 (0.05) |
| **LYD** | conv | 0.17 (0.08) | 0.2 (0.06) |
| **CLD** | conv | 0.07 (0.06) | 0.1 (0.06) |
| **CBF** | conv | 0.14 (0.07) | 0.2 (0.06) |

Supplemental Table 23. Estimates of the proportion of phenotypic variance explained by omics relationship matrices ($\hat{h}^{2}$) and litter effects ($c^{2}$) for the TPMBLUP (mean) model.

| **Trait** | **converge** | **h^2^_TPM_mean_ (SE)** | **c^2^ (SE)** |
| --- | --- | --- | --- |
| **qNurADG** | conv | 0.27 (0.06) | 0.17 (0.04) |
| **qNurHS1** | conv | 0.1 (0.04) | 0 (0.04) |
| **qNurHS2** | conv | 0.07 (0.03) | 0.06 (0.05) |
| **cNurADG** | conv | 0.04 (0.03) | 0.11 (0.04) |
| **cNurHS** | conv | 0.04 (0.03) | 0.03 (0.04) |
| **cNurTRT** | conv | 0 (0.01) | 0.04 (0.04) |
| **cNurMOR** | conv | 0.01 (0.01) | 0 (0) |
| **cFinADG** | conv | 0.02 (0.03) | 0.18 (0.05) |
| **cFinHS** | conv | 0.02 (0.02) | 0.06 (0.05) |
| **cFinTRT** | conv | 0 (0) | 0.05 (0.05) |
| **AllTRT** | conv | 0 (0) | 0.03 (0.05) |
| **cFinMOR** | conv | 0.01 (0.02) | 0 (0) |
| **AllMOR** | conv | 0.02 (0.02) | 0 (0) |
| **ADFI** | conv | 0.05 (0.04) | 0.16 (0.05) |
| **FCR** | conv | 0.09 (0.04) | 0.25 (0.05) |
| **RFI** | conv | 0.01 (0.02) | 0.13 (0.05) |
| **CWT** | conv | 0.01 (0.03) | 0.28 (0.05) |
| **DRS** | conv | 0 (0) | 0.21 (0.05) |
| **LYD** | conv | 0.06 (0.04) | 0.25 (0.06) |
| **CLD** | conv | 0.03 (0.04) | 0.12 (0.06) |
| **CBF** | conv | 0.06 (0.04) | 0.25 (0.06) |

Supplemental Table 24. Estimates of the proportion of phenotypic variance explained by omics relationship matrices ($\hat{h}^{2}$) and litter effects ($c^{2}$) for the GTPMBLUP model.

| **Trait** | **converge** | **h^2^_G_ (SE)** | **h^2^_T_ (SE)** | **h^2^_P_ (SE)** | **h^2^_M_ (SE)** | **h^2^_GTPM_ (SE)** | **c^2^ (SE)** |
| --- | --- | --- | --- | --- | --- | --- | --- |
| **qNurADG** | conv | 0.09 (0.08) | 0.03 (0.03) | 0.1 (0.05) | 0.1 (0.03) | 0.32 (0.09) | 0.14 (0.05) |
| **qNurHS1** | Not conv |  |  |  |  |  |  |
| **qNurHS2** | conv | 0.06 (0.08) | 0 (0) | 0.01 (0.01) | 0.06 (0.03) | 0.12 (0.09) | 0.06 (0.05) |
| **cNurADG** | Not conv |  |  |  |  |  |  |
| **cNurHS** | Not conv |  |  |  |  |  |  |
| **cNurTRT** | conv | 0.18 (0.08) | 0.01 (0.01) | 0 (0.01) | 0 (0) | 0.18 (0.08) | 0 (0.04) |
| **cNurMOR** | conv | 0.14 (0.08) | 0 (0) | 0 (0) | 0 (0) | 0.16 (0.08) | 0.24 (0.15) |
| **cFinADG** | conv | 0.2 (0.09) | 0 (0.01) | 0 (0) | 0.01 (0.01) | 0.21 (0.09) | 0.12 (0.06) |
| **cFinHS** | conv | 0.01 (0.07) | 0.52 (0.08) | 0.01 (0.02) | 0 (0.01) | 0.02 (0.08) | 0.06 (0.05) |
| **cFinTRT** | conv | 0 (0) | 0.01 (0.02) | 0 (0) | 0 (0) | 0.01 (0.02) | 0.05 (0.05) |
| **AllTRT** | Not conv |  |  |  |  |  |  |
| **cFinMOR** | conv | 0 (0) | 0 (0.01) | 0 (0) | 0.01 (0.02) | 0.02 (0.02) | 0 (0) |
| **AllMOR** | Not conv |  |  |  |  |  |  |
| **ADFI** | conv | 0.36 (0.11) | 0 (0) | 0 (0) | 0.02 (0.02) | 0.39 (0.11) | 0.05 (0.05) |
| **FCR** | conv | 0.34 (0.1) | 0 (0) | 0.03 (0.03) | 0.02 (0.01) | 0.47 (0.11) | 0.18 (0.06) |
| **RFI** | conv | 0.68 (0.09) | 0 (0) | 0 (0.01) | 0 (0) | 0.68 (0.09) | 0 (0) |
| **CWT** | conv | 0.05 (0.08) | 0.72 (0.11) | 0.02 (0.01) | 0 (0) | 0.77 (0.11) | 0.22 (0.01) |
| **DRS** | Not conv |  |  |  |  |  |  |
| **LYD** | Not conv |  |  |  |  |  |  |
| **CLD** | conv | 0.42 (0.11) | 0 (0) | 0 (0.01) | 0.01 (0.02) | 0.45 (0.11) | 0 (0) |
| **CBF** | conv | 0.7 (0.11) | 0 (0) | 0.01 (0.02) | 0.02 (0.02) | 0.75 (0.11) | 0.02 (0.05) |

Supplemental Table 25. Estimates of the proportion of phenotypic variance explained by omics relationship matrices ($\hat{h}^{2}$) and litter effects ($c^{2}$) for the GTPMBLUP (mean) model.

| **Trait** | **converge** | **h^2^_GTPM_mean_ (SE)** | **c^2^ (SE)** |
| --- | --- | --- | --- |
| **qNurADG** | conv | 0.35 (0.07) | 0.14 (0.04) |
| **qNurHS1** | conv | 0.13 (0.05) | 0 (0) |
| **qNurHS2** | conv | 0.1 (0.04) | 0.06 (0.05) |
| **cNurADG** | conv | 0.09 (0.05) | 0.1 (0.04) |
| **cNurHS** | conv | 0.06 (0.04) | 0.03 (0.04) |
| **cNurTRT** | conv | 0.01 (0.02) | 0.04 (0.04) |
| **cNurMOR** | conv | 0.01 (0.02) | 0 (0) |
| **cFinADG** | conv | 0.04 (0.04) | 0.18 (0.05) |
| **cFinHS** | conv | 0.03 (0.03) | 0.06 (0.05) |
| **cFinTRT** | conv | 0 (0) | 0.05 (0.05) |
| **AllTRT** | conv | 0 (0) | 0.03 (0.05) |
| **cFinMOR** | conv | 0.01 (0.03) | 0 (0) |
| **AllMOR** | conv | 0.03 (0.03) | 0 (0) |
| **ADFI** | conv | 0.11 (0.06) | 0.14 (0.05) |
| **FCR** | conv | 0.17 (0.07) | 0.24 (0.05) |
| **RFI** | conv | 0.09 (0.06) | 0.11 (0.05) |
| **CWT** | conv | 0.03 (0.04) | 0.27 (0.05) |
| **DRS** | conv | 0 (0) | 0.21 (0.05) |
| **LYD** | conv | 0.2 (0.08) | 0.2 (0.06) |
| **CLD** | conv | 0.09 (0.06) | 0.1 (0.06) |
| **CBF** | conv | 0.16 (0.08) | 0.21 (0.06) |

Supplemental Table 26. Estimates of the phenotypic prediction accuracy based on 26 different combination of different omics relationship matrices (genomics, G; transcriptomics, T; proteomics, P; metabolomics, M) for each performance and disease resilience trait.

|  | **G** | **T** | **P** | **M** | **GT** | **GT (mean)** | **GP** | **GP (mean)** | **GM** | **GM (mean)** | **TP** | **TP (mean)** | **TM** | **TM (mean)** | **PM** | **PM (mean)** | **GTP** | **GTP (mean)** | **GTM** | **GTM (mean)** | **GPM** | **GPM (mean)** | **TPM** | **TPM (mean)** | **GTPM** | **GTPM (mean)** |
| --- | --- | --- | --- | --- | --- | --- | --- | --- | --- | --- | --- | --- | --- | --- | --- | --- | --- | --- | --- | --- | --- | --- | --- | --- | --- | --- |
| **qNurADG** | 0.029 | 0.320 | 0.272 | 0.493 | 0.291 | 0.296 | 0.146 | 0.265 | 0.470 | 0.472 | 0.295 | 0.340 | 0.490 | 0.490 | 0.457 | 0.487 | NA | 0.330 | 0.551 | 0.473 | 0.422 | 0.469 | NA | 0.488 | 0.145 | 0.473 |
| **qNurHS1** | 0.052 | 0.108 | 0.146 | 0.221 | 0.105 | 0.106 | 0.055 | 0.124 | 0.217 | 0.222 | 0.116 | 0.140 | 0.191 | 0.205 | NA | 0.221 | 0.119 | 0.138 | 0.325 | 0.207 | 0.265 | 0.222 | NA | 0.208 | 0.051 | 0.208 |
| **qNurHS2** | 0.026 | 0.182 | 0.153 | 0.329 | 0.186 | 0.194 | 0.024 | 0.127 | 0.326 | 0.330 | 0.162 | 0.173 | 0.329 | 0.333 | 0.353 | 0.300 | NA | 0.177 | 0.416 | 0.334 | 0.320 | 0.303 | NA | 0.310 | 0.074 | 0.313 |
| **cNurADG** | 0.172 | 0.101 | 0.048 | 0.146 | 0.187 | 0.193 | NA | 0.180 | 0.205 | 0.177 | 0.007 | 0.107 | 0.138 | 0.155 | NA | 0.151 | 0.072 | 0.193 | 0.409 | 0.184 | NA | 0.176 | NA | 0.156 | 0.166 | 0.182 |
| **cNurHS** | 0.066 | 0.061 | 0.081 | 0.127 | 0.014 | 0.079 | 0.079 | 0.092 | 0.127 | 0.139 | 0.079 | 0.079 | 0.125 | 0.136 | 0.112 | 0.211 | NA | 0.090 | NA | 0.144 | 0.098 | 0.128 | NA | 0.128 | 0.094 | 0.134 |
| **cNurTRT** | 0.093 | 0.053 | -0.036 | 0.015 | 0.085 | 0.121 | 0.104 | 0.047 | 0.091 | 0.041 | 0.063 | 0.016 | 0.007 | 0.029 | -0.050 | 0.012 | -0.002 | 0.038 | 0.356 | 0.047 | 0.001 | 0.028 | NA | 0.016 | 0.065 | 0.030 |
| **cNurMOR** | 0.042 | 0.132 | 0.036 | 0.041 | 0.076 | 0.137 | NA | 0.041 | 0.041 | 0.045 | 0.062 | 0.064 | 0.121 | 0.094 | -0.016 | 0.031 | 0.066 | 0.072 | 0.308 | 0.096 | 0.013 | 0.038 | NA | 0.060 | 0.146 | 0.063 |
| **cFinADG** | 0.125 | -0.028 | 0.011 | 0.116 | 0.125 | 0.039 | -0.063 | 0.050 | NA | 0.134 | NA | -0.017 | 0.123 | 0.099 | 0.088 | NA | 0.109 | 0.000 | 0.343 | 0.120 | 0.154 | 0.111 | NA | 0.084 | 0.046 | 0.102 |
| **cFinHS** | 0.038 | 0.012 | 0.038 | 0.002 | -0.027 | 0.018 | NA | 0.037 | -0.062 | 0.003 | -0.037 | 0.037 | 0.039 | 0.011 | 0.002 | 0.017 | NA | 0.035 | 0.290 | 0.014 | -0.085 | 0.019 | NA | 0.019 | 0.027 | 0.021 |
| **cFinTRT** | 0.023 | 0.063 | -0.054 | -0.068 | 0.063 | 0.061 | 0.028 | -0.052 | NA | -0.080 | NA | -0.028 | 0.068 | -0.033 | -0.156 | NA | 0.083 | -0.025 | 0.066 | -0.031 | -0.009 | -0.098 | NA | -0.069 | 0.076 | -0.068 |
| **AllTRT** | 0.153 | 0.047 | -0.061 | -0.013 | 0.118 | 0.107 | 0.109 | -0.019 | 0.169 | -0.013 | -0.063 | -0.026 | -0.118 | -0.021 | -0.033 | -0.048 | 0.123 | -0.014 | NA | -0.005 | 0.107 | -0.042 | NA | -0.044 | 0.070 | -0.034 |
| **cFinMOR** | -0.003 | 0.030 | -0.060 | 0.050 | 0.030 | 0.032 | -0.069 | -0.074 | 0.083 | 0.071 | -0.011 | -0.035 | 0.037 | 0.049 | 0.027 | -0.030 | 0.049 | -0.034 | 0.114 | 0.049 | 0.037 | -0.005 | NA | 0.010 | 0.000 | 0.007 |
| **AllMOR** | 0.052 | 0.108 | -0.006 | 0.063 | 0.092 | 0.109 | 0.060 | -0.002 | 0.048 | 0.071 | 0.077 | 0.038 | 0.104 | 0.102 | 0.001 | 0.003 | NA | 0.044 | 0.278 | 0.106 | -0.009 | 0.015 | NA | 0.057 | 0.082 | 0.060 |
| **ADFI** | 0.173 | 0.056 | 0.030 | 0.185 | 0.168 | 0.155 | 0.094 | 0.090 | NA | 0.203 | 0.035 | 0.035 | 0.156 | 0.193 | 0.164 | NA | NA | 0.088 | 0.419 | 0.213 | 0.160 | 0.175 | NA | 0.170 | 0.148 | 0.184 |
| **FCR** | 0.214 | 0.230 | 0.070 | 0.129 | 0.293 | 0.298 | 0.169 | 0.187 | NA | 0.142 | NA | 0.220 | 0.235 | 0.207 | 0.212 | 0.154 | 0.245 | 0.283 | 0.449 | 0.226 | 0.205 | 0.168 | NA | 0.219 | 0.253 | 0.235 |
| **RFI** | 0.149 | 0.081 | 0.059 | -0.015 | 0.147 | 0.152 | 0.050 | 0.056 | NA | NA | NA | 0.056 | -0.055 | 0.006 | NA | -0.038 | 0.181 | NA | 0.120 | 0.046 | 0.148 | 0.057 | NA | 0.028 | 0.142 | 0.065 |
| **CWT** | 0.085 | 0.137 | -0.010 | 0.073 | NA | 0.133 | NA | -0.019 | NA | 0.092 | NA | 0.003 | NA | 0.067 | 0.014 | 0.008 | NA | -0.115 | NA | 0.106 | 0.000 | 0.034 | NA | 0.020 | 0.140 | 0.023 |
| **HHB** | 0.098 | -0.028 | 0.022 | 0.097 | 0.098 | -0.001 | 0.170 | 0.011 | 0.120 | 0.072 | -0.049 | 0.005 | -0.042 | 0.032 | NA | 0.000 | 0.023 | 0.011 | 0.345 | 0.039 | NA | 0.017 | NA | 0.008 | 0.112 | 0.013 |
| **LYD** | 0.282 | 0.195 | 0.172 | 0.046 | 0.323 | 0.320 | NA | 0.287 | NA | NA | 0.194 | 0.231 | 0.079 | 0.142 | 0.138 | 0.182 | 0.399 | 0.325 | 0.604 | NA | 0.375 | 0.184 | NA | 0.190 | 0.204 | 0.231 |
| **CLD** | 0.241 | -0.039 | 0.039 | 0.063 | 0.207 | 0.157 | 0.048 | 0.093 | 0.311 | 0.122 | -0.171 | -0.022 | -0.130 | 0.010 | -0.076 | 0.013 | 0.324 | 0.046 | NA | 0.075 | 0.305 | 0.108 | NA | 0.030 | 0.080 | 0.064 |
| **CBF** | 0.296 | 0.203 | 0.144 | 0.071 | 0.332 | 0.312 | 0.242 | 0.275 | 0.241 | NA | 0.190 | 0.218 | 0.092 | 0.154 | 0.111 | 0.130 | 0.432 | 0.310 | NA | 0.199 | 0.486 | 0.182 | NA | 0.195 | 0.200 | 0.230 |

Supplemental Table 27. Estimates of the AUC for mortality prediction using 26 combinations of omics relationship matrices (genomics, G; transcriptomics, T; proteomics, P; metabolomics, M).

|  | **G** | **T** | **P** | **M** | **GT** | **GT (mean)** | **GP** | **GP (mean)** | **GM** | **GM (mean)** | **TP** | **TP (mean)** | **TM** | **TM (mean)** | **PM** | **PM (mean)** | **GTP** | **GTP (mean)** | **GTM** | **GTM (mean)** | **GPM** | **GPM (mean)** | **TPM** | **TPM (mean)** | **GTPM** | **GTPM (mean)** |
| --- | --- | --- | --- | --- | --- | --- | --- | --- | --- | --- | --- | --- | --- | --- | --- | --- | --- | --- | --- | --- | --- | --- | --- | --- | --- | --- |
| **cNurMOR** | 0.561 | 0.563 | 0.417 | 0.504 | 0.562 | 0.576 | NA | 0.429 | 0.561 | 0.502 | 0.559 | 0.495 | 0.562 | 0.523 | 0.400 | 0.468 | 0.531 | 0.505 | 0.662 | 0.537 | 0.517 | 0.467 | NA | 0.500 | 0.541 | 0.501 |
| **cFinMOR** | 0.483 | 0.476 | 0.455 | 0.536 | 0.476 | 0.475 | 0.415 | 0.437 | 0.554 | 0.530 | 0.471 | 0.447 | 0.544 | 0.512 | 0.497 | 0.502 | 0.477 | 0.452 | 0.560 | 0.507 | 0.450 | 0.512 | NA | 0.477 | 0.511 | 0.476 |
| **AllMOR** | 0.516 | 0.537 | 0.478 | 0.526 | 0.533 | 0.540 | 0.536 | 0.472 | 0.519 | 0.537 | 0.564 | 0.497 | 0.538 | 0.552 | 0.497 | 0.470 | NA | 0.503 | 0.647 | 0.555 | 0.496 | 0.464 | NA | 0.523 | 0.499 | 0.527 |
